# Teachers’ and students’ use of ChatGPT at Social science faculty in the public and private Universities of Bangladesh

**DOI:** 10.12688/f1000research.161351.1

**Published:** 2025-03-06

**Authors:** Arifur Rahman, Md Khairul Islam, Abdullah Al-Mamun, Md Shahidul Islam

**Affiliations:** 1 Institute of Education and Research, University of Dhaka, University of Dhaka, Dhaka, Dhaka, 1000, Bangladesh; 2 Institute of Education and Research, University of Dhaka, Dhaka, Dhaka, 1000, Bangladesh; 3 Institute of Education and Research, University of Dhaka, Dhaka, Dhaka, 1000, Bangladesh; 4 Jaggo Foundation, Rajshahi, Rajshahi, 6205, Bangladesh

**Keywords:** ChatGPT, teaching-learning, perception, academic integrity, reliability, ethical consideration, higher education

## Abstract

**Background:**

Bangladesh is an emerging country where teachers and students of public and private universities have started using technology in the classrooms. Many teachers and students of social science faculty have an inclination to use ChatGPT for educational and research purposes. By focusing on this specific context, the study aims to bring insights into the perception and integration of ChatGPT into the educational practices in an emerging country.

**Methods:**

This study employed a mixed method approach. Quantitative data were collected through questionnaire survey from 402 teachers and 440 students of eight different public and private universities following a stratified sampling approach. A convenience sampling technique was followed with a view to collecting qualitative data through in-depth interviews of 32 participants, including 16 teachers and 16 students from both public and private universities.

**Results:**

The research presents that students and teachers both have proficiency, but there is a gap in expertise. Students perceive ChatGPT as beneficial for better learning outcomes, and teachers find it helpful in preparing for classes and instructional materials. Both teachers and students consider ChatGPT requirng minimal effort. Though students are influenced by their peers to use it, teachers are not. On the other hand, teachers have more behavioral intentions to use it in the future than the students have. Yet worries over ethical use, reliance, and information accuracy prevail. High cost and language barriers are also listed as reasons for level of accessibility .

**Conclusion:**

The findings of this study have significant implications for the development of policies, research endeavors, and teaching-learning practices in the higher education sector covering both public and private universities in Bangladesh and similar contexts.

## Introduction

ChatGPT, an AI technology bearding the GPT (Generative Pre-trained Transformer) architecture, can reasonably improve the teaching and learning process due to its sage conversational capabilities ( Baidoo-Anu & Ansah, 2023). It can provide translation, summary, and question answers, and simultaneously, the generation of texts automates pestering ( Cotton et al., 2023). Unsupervised pre-training and controlled fine-tuning generate responses similar to what a human expert would say, displaying domain knowledge about various topics and answering questions accordingly. Textual data is gathered from various internet sources, including websites, articles, papers, and forums ( Dwivedi et al., 2023). The most notable feature of ChatGPT is its ability to generate text. Within just three months of the collective release of ChatGPT, significant numbers of software engineers, authors, academics, teachers, and songwriters used the system to generate written content, academic essays, computer programs, applications, and song lyrics ( Mariani et al., 2023). ChatGPT-4 has continued to be developed, and today, it is used across different applications, including education worldwide. It also helps promoting students' educational experiences through tailored coaching and support. Implementing this technology has advanced student engagement and collaboration by allowing students to question and debate asynchronously, removing the need to be simultaneously present ( Li & Xing, 2021). ChatGPT can help teachers and students discuss complex ideas at their desired speed. It also helps with administrative tasks such as responding to commonly asked questions, providing course materials, and managing scheduling or registration tasks. Universities can utilize ChatGPT to improve student engagement by providing continuous support and accelerating administrative processes ( Biswas, 2023).

However, despite these advantages, some important issues must be remembered before utilizing ChatGPT. When it is iused for educational assessment, the concern of plagiarism arises. AI essay-writing systems produce essays based on predetermined parameters or guiding principles. They can commit academic fraud forgery by submitting plagiarized content write-ups. It counters the key central idea of education, which is to educate and inspire learners, and it could undermine the credibility of academic degrees in the coming years ( Dehouche, 2021). So, the role of ChatGPT in education has sparked and initiated one of the most talked about debates in academia. Further research is needed to determine ChatGPT adoption in developed and developing countries. An in-depth analysis shows that comparatively, more affluent countries are moving quickly on the chatbots issue and developing ways to leverage it for education. However, developing countries such as Bangladesh need to focus on this. Because technologies do not have borders, understanding the utilization of ChatGPT by a diverse country like Bangladesh could provide a worldwide perspective of the benefits and challenges of adopting and adapting AI technologies in multiple socio-cultural and economic environments. Bangladesh is a developing country where the educational system is being modernized, and technology is being introduced in the classrooms of public and private universities ( Sarker et al., 2019).

Recently, many tertiary level teachers and students of Bangladesh thave been found interested in using ChatGPT. By focusing on this specific context, the study aims to bring insight into the perception and integration of emerging advanced technology such as ChatGPT into the educational practices of an emerging country. Focusing on Bangladesh as a specific research context, the study aspires to produce meaningful and enlightening knowledge for the worldwide community. In the backdrop of social science faculty at the public and private universities of Bangladesh, where teaching and personalized learning pathways are of great importance, it is essential to address teachers' and students' use of ChatGPT. Comparatively, the educational system of Bangladesh has its drawbacks, with large classroom setups, shortage of resources, and potentially low student participation ( Prodhan, 2016). These issues may be mitigated through ChatGPT, which provides personalized support, immediate feedback, and a highly interactive learning environment. Understanding key stakeholders' perceptions and the use of ChatGPT is crucial to harness this technology effectively. Under this circumstance, getting insight into teachers' and students' perspectives on employing ChatGPT cannot be neglected because they are an indispensable part of the teaching-learning process.

There is a lack of knowledge about the perceptions and use of ChatGPT among teachers and students in the general settings of social science faculty at public and private universities in Bangladesh. This study aims to fill this knowledge gap. In order to comprehensively evaluate the potential and challenges of implementing ChatGPT in the teaching-learning process in developing countries such as Bangladesh, it is crucial to gain insights into teachers' and students' perceptions and usage of ChatGPT. Understanding the perspectives and usage of ChatGPT by teachers and students at social science faculty in developing nations on ChatGPT is essential to ensure its implementation aligns with their specific needs and circumstances. Thus, the objective of this study is to investigate the perceptions and usage of ChatGPT by teachers and students of the social science faculty at public and private universities in Bangladesh.

## Theoretical framework

**Figure 1.**
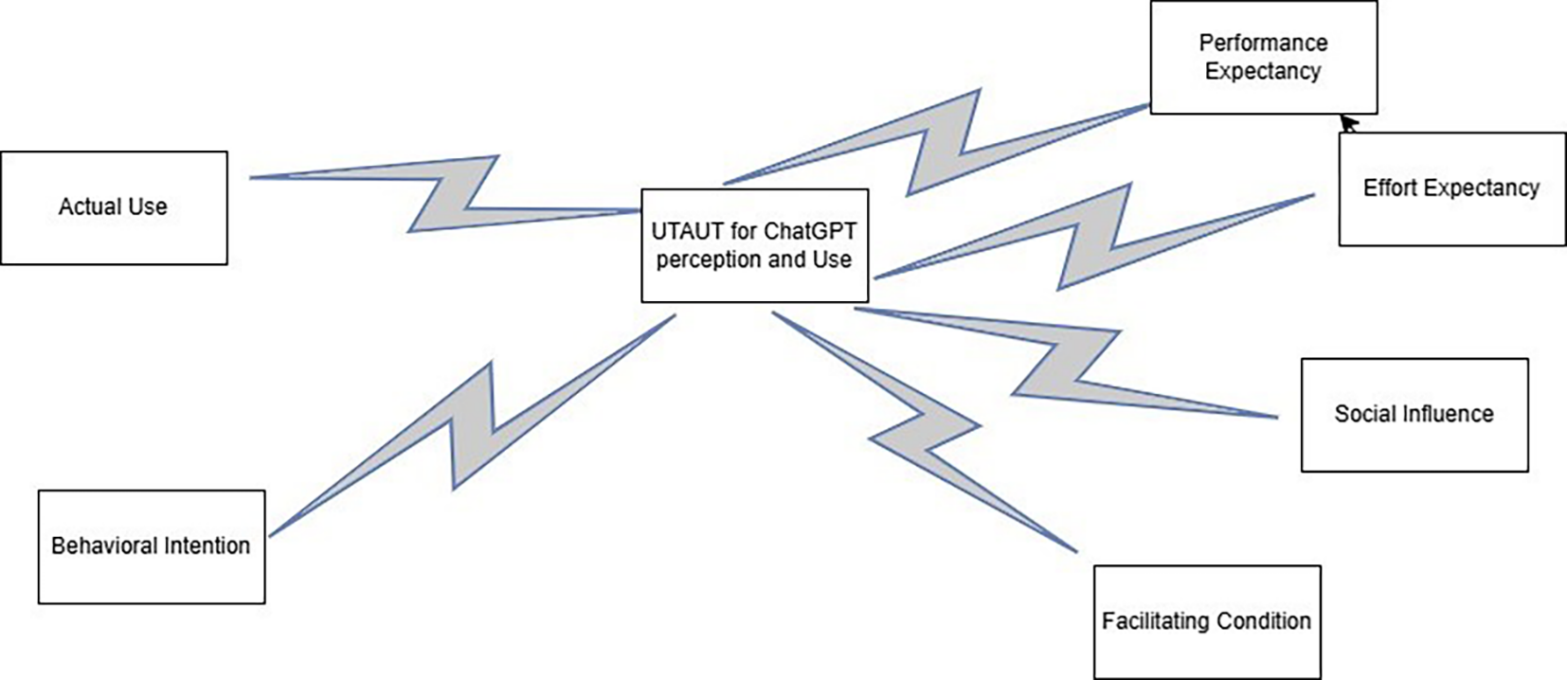
Theoretical framework of this study. This figure delineates the theoretical framework that guides this study. This framework is based on the Unified Theory of Acceptance and Use of Technology (UTAUT) model proposed by Venkatesh et al. (2003).

This study is grounded in the Unified Theory of Acceptance and Use of Technology (UTAUT) model, which posits four core constructs such as performance expectancy, effort expectancy, social influence, and facilitating conditions that are theorized to shape the acceptance and usage of technology ( Venkatesh et al., 2003). Performance expectancy is defined as the extent to which ChatGPT is perceived to improve educational outcomes, such as improving teaching efficiency for teachers or helping students during the learning process. Effort expectancy answers how easily teachers and students can leverage ChatGPT effectively during their studies. The social influence reflects the effect of peers, faculty, and institutional norms on the decisions of individuals to adopt ChatGPT. Finally, facilitating conditions are the resources and support at the educational institution that enable the use of ChatGPT, such as training or technical support. Along with these constructs, the UTAUT model added two vital outcomes: behavioral intention and actual use ( Rofiah & Suhermin, 2022). The teachers’ and students’ perceptions and how they use ChatGPT were guided by behavioral intention and actual use outcomes to fulfill the objectives of this study ( Figure 1).

## Methods

This study followed a mixed method approach combining both quantitative and qualitative data to get a comprehensive understanding of teachers’ and students’ perception and usage of ChatGPT in social science faculty of both public and private unviersities in Bangladesh. This approach allow the researchers to generalize the perception and usage of ChatGPT by teachers and students along with the exploration of underlying views, experiences, and contextual factors of adapting ChatGPT. Heyvaert et al. (2013) argue that mixed-methods research is crucial because it combines quantitative data with qualitative data, which may provide a holistic understanding of complex phenomena. This strategy allows for a more in-depth exploration of findings. As a result, this study followed a mixed-method approach to generalize social science faculty teachers' and students' perceptions and use of ChatGPT in Bangladesh and also to comprehensively get an in-depth scenario of their experiences, strategies, and interactions. Two survey questionnaires were generated for both teachers and students. Each survey question contains twenty-six items and is prepared on a five-point Likert scale. In order to gain a comprehensive understanding of the perspectives of teachers and students from public and private universities in Bangladesh, interviews were carried out. As a theoretical framework, the UTAUT model helps to guide the preparation of questionnaires and the interpretation of the findings. However, no copyrighted survey items or scales from the proprietary UTAUT instrument were used in this study; rather, the model was adapted conceptually to develop data collection tools and also cited properly.

Forty teachers and fifty-three students participated in the piloting of two surveys. While the students' survey had a Cronbach's alpha of 0.833, the teachers' survey had a Cronbach's alpha of 0.877. These results show that both questionnaires are highly reliable for gathering data. A stratified sampling technique was used to collect online survey data. In terms of sample size, data were collected from the social science faculties of sixteen universities, including eight public and eight private universities. In total, 402 teachers and 440 students' survey data were collected from these social science faculties, though after data cleaning, 381 teachers' data and 384 students' data were prevailed. Gender and other demographic issues were considered to collect data. Then data was analyzed through IBM SPSSversion 27 ( IBM Corp, 2021) ( https://www.ibm.com/support/pages/downloading-ibm-spss-statistics-27). For researchers who seek an open-source alternative, can use, JASP for similar data analysis. JASP is an open-access software (available at https://jasp-stats.org/).

In terms of qualitative data, a convenient sampling technique was used for conducting in-depth interviews. A total of 32 interviews were conducted, involving 16 teachers and 16 students. Two teachers, and two students from each university were selected. The interviews were recorded with permission.

Research objectives were explained to the participants and commitment of conserving privacy of the participants in every aspects of the research were declared. Interviews were taken only those who willingly showed interest to participate in the study. Each interview lasted for 35 to 40 minutes. After collecting data, every recorded interview was transcribed verbatim. Next, all Bengali transcriptions were converted into English and was sent these to a Bengali and English language specialist to ensure the accuracy of the translation.

Researchers completed transcriptions after getting the translator's input. The research employed the thematic qualitative data analysis technique to identify patterns and theme to understand the real situation deeply ( Braun & Clarke, 2006). In accordance with the six stages of data analysis outlined by Braun and Clarke (2006), researchers first became acquainted with the data before creating preliminary codes from it. At this point, they adopted the strategy of “reading, reading and reading again” ( Mertler, 2009, p. 141) to examine all narrative data. Researchers looked for new themes in the third step, and then went over the themes again in the fourth. At the fifth step, many themes were identified and given names, and a report was ultimately generated. Since researchers moved from the facts to a theoretical understanding, they used an inductive approach to qualitative data analysis ( Graneheim, Lindgren, & Lundman, 2017).

In order to assure the dependability of analysis and reporting conclusions, researchers considered the criterion of utilizing low-inference descriptors ( Silverman, 2011). According to Johnson (1997), low-inference descriptors refer to the practice of using descriptions that closely resemble the accounts provided by participants and the field notes of researchers. Direct quotations, known as verbatim, are frequently utilized as a low inference descriptor. Considering this approach, researchers diligently transcribed the recordings and incorporated substantial data samples into the research conclusions ( Johnson, 1997; Silverman, 2011).

## Findings

The following ( Table 1) demonstrates the demographic information of the teachers.

**Table 1.** Teachers demographic information.

		Count	Table N %
Types of University	Public University	202	53.0%
	Private University	179	47.0%
Gender	Male	207	54.3%
	Female	174	45.7%
	Others	0	0.0%
	No interest to share	0	0.0%
Position	Professor	39	10.2%
	Associate professor	62	16.3%
	Assistant professor	148	38.8%
	Lecturer	132	34.6%
Department	Economics	99	26.0%
	Political Science	80	21.0%
	Sociology	22	5.8%
	International Relationship	31	8.1%
	Mass Communication & Journalism	56	14.7%
	Public Administration	56	14.7%
	Anthropology	8	2.1%
	Population Science	16	4.2%
	Folklore	9	2.4%
	Social Work	4	1.0%
ChatGPT	Free version	302	79.3%
	Paid version	79	20.7%

Regarding the demography of teachers , it was found that public universities represented the majority of participants that is 53% of the 381 participants, while private universities represented 47%. In terms of gender, male participants accounted for 54% and female for 46%. Academically, assistant professors led by 38.8%, followed by lecturers of 34.6%, while associate professors and full professors made up 16.3% and 10.2%, respectively. Breaking it down by department, results found Economics to be the most represented discipline of 26%, followed by Political Science of 21%. Moreover, Mass Communication and Journalism, and Public Administration each accounted for 14.7% of the sample, and participants from the Anthropology department accounted for only 1%. The majorities, 79.3% of teachers, have a free version of ChatGPT, and 20.7% have a premium version.

The following table ( Table 2) demonstrates the demographic information of the students.

**Table 2.** Student demographic information.

		Count	Table N %
Types of University	Public University	213	55.5%
	Private University	171	44.5%
Gender	Male	213	55.5%
	Female	171	44.5%
	Others	0	0.0%
	No interest to share	0	0.0%
Education level	Graduate	206	53.6%
	Post Graduate	178	46.4%
Education year	1st year	33	8.6%
	2nd year	82	21.4%
	3rd year	98	25.5%
	4th year	69	18.0%
	Masters 1st Semester	45	11.7%
	Masters 2nd Semester	36	9.4%
	Master’s Thesis Student	21	5.5%
Subject of Study	Economics	21	5.5%
	Political Science	34	8.9%
	Sociology	28	7.3%
	International Relationship	41	10.7%
	Mass Communication & Journalism	49	12.8%
	Public Administration	40	10.4%
	Anthropology	25	6.5%
	Population Science	25	6.5%
	Peace and Conflict	26	6.8%
	Social Work	27	7.0%
	Criminology	14	3.6%
	Women and Gender Studies	14	3.6%
	Development Studies	6	1.6%
	Television, Film and Photography	11	2.9%
	Education	22	5.7%
	Others	1	0.3%
Device	Laptop	192	50.0%
	Computer	58	15.1%
	Smartphone	131	34.1%
	Tab	3	0.8%
	Others	0	0.0%
ChatGPT	Free version	372	97.1%
	Paid version	11	2.9%

From the demography of students, it has been found that 55.5% were from public universities and 44.5% from private universities. The study includes the same gender distribution, with males constituting 55.5% and females 44.5%. Regarding the academic level, 53.6% of the respondents were graduate students, and 46.4% were post-graduate students. Mass Communication and Journalism was the most represented department in this study with 12.8% of students, followed by International Relationships (10.7%) and Public Administration (10.4%). Development Studies was the least represented department with1.6%. As for the usage of ChatGPT, 97.1% students utilized the free version, and merely 2.9% were premium users.

Teachers’ responses to the survey are presented in Table 3.

**Table 3.** Teacher response to the survey items.

Items	Mean	Std. Deviation
**Performance Expectancy (PE)**		
ChatGPT improves my capacity to teach complex issues efficiently	3.48	.803
Using ChatGPT enhances my efficiency in developing lesson plans and lectures	3.37	.796
ChatGPT assists me in developing more interesting teaching materials	3.51	.807
ChatGPT facilitates my research process, which encompasses concept formulation, literature review, methodology, and report writing	2.91	.858
ChatGPT enhances the quality of my study by providing rapid access to relevant materials	3.30	.937
**Effort Expectancy (EE)**		
I find that ChatGPT is user-friendly when developing instructional materials	3.58	.809
Incorporating ChatGPT with my teaching approaches requires minimal effort	3.48	.841
Understanding the use of ChatGPT for research activities is simple	2.83	1.024
I am confident of my ability to use ChatGPT for teaching and research	2.80	.955
**Social Influence (SI)**		
My colleagues support the use of ChatGPT in my teaching and research effort	2.69	.929
My institution supports the integration of ChatGPT within the educational environment	2.67	.904
The use of ChatGPT is becoming increasingly popular among teachers at my university	3.46	.944
**Facilitating Condition (FC)**		
I have the required technological resources to use ChatGPT in my teaching and research activities	3.32	1.142
My university offers adequate support for using ChatGPT as a teaching and research facility	2.49	.996
Institutional policies and guidelines for the ethical use of ChatGPT in educational contexts are established	2.47	1.017
I have financial support for using the Premium edition of ChatGPT	2.42	1.075
**Behavioral Intention (BI)**		
I plan to continue using ChatGPT for my teaching and research activities	3.62	1.023
I plan to increase my use of ChatGPT in the future for both teaching and research aims	3.68	.967
**Actual Use (AU)**		
I frequently utilize ChatGPT to develop teaching materials and lesson plans	3.73	1.089
I frequently utilize ChatGPT to assist with my research activities	3.74	.934
I frequently utilize ChatGPT to assign task to the students	2.87	1.080
I frequently consider the risk of plagiarism and ethical issues when using ChatGPT in my teaching and research	3.59	.944
I frequently verify the accuracy of the information supplied by ChatGPT	3.88	.931
I actively seek training opportunities to improve my use of ChatGPT for teaching and research	3.81	1.043
I am cautious about relying too heavily on ChatGPT for creativity in my teaching and research	3.82	1.015
I am cautious about potential challenges of using ChatGPT	3.70	.959

Teachers considered ChatGPT positively regarding its effective integration into enhancing teaching materials (M=3.51, SD=0.807), easy use (M=3.58, SD=0.809), and less effort required for incorporation into the teaching methods (M=3.48, SD=0.841). ChatGPT was frequently used to generate teaching materials (M=3.73, SD=1.089) and to check the accuracy of information (M=3.88, SD=0.931) but rated lower for helping with complex research (e.g., literature reviews and methods) (M=2.91, SD=0.858). The confidence level for using ChatGPT for research was relatively low (M=2.80, SD=0.955). Teachers also reported low levels of institutional support (M=2.49, SD=0.996) and policy guidance for ethical use (M=2.47, SD=1.017). Similarly, colleagues' support (M=2.69, SD=0.929) and financial support for premium usage (M=2.42, SD=1.075) were presented as insufficient. Teachers intended to continue using ChatGPT (M=3.62, SD=1.023) and use it more (M=3.68, SD=0.967). They often thought about ethical risks (M=3.59, SD=0.944) and accuracy verification (M=3.88, SD=0.931), indicating a cautious optimism. They proactively sought training opportunities (M=3.81, SD=1.043) and were careful of excessive reliance on ChatGPT for creativity (M=3.82, SD=1.015).

Students’ response to the items is shown in Table 4.

**Table 4.** Student response to items.

Items	Mean	Std. Deviation
**Performance Expectancy (PE)**		
ChatGPT enhances my capacity to complete assignments more efficiently	4.31	.591
Applying ChatGPT enhances my understanding of complicated subjects and concepts	4.47	.625
ChatGPT improves the quality of my research papers and essays	4.04	.601
ChatGPT assists me in generating concepts for my research projects and assignments	4.44	.769
Employing ChatGPT enables me to accomplish work with greater rapidity and accuracy	4.47	.729
**Effort Expectancy (EE)**		
ChatGPT helps to finish my projects easily	4.05	.813
I can quickly acquire proficiency in utilizing ChatGPT for my research activities	4.21	.755
Using ChatGPT for academic study and research requires minimal effort	4.15	1.051
I am competent in utilizing ChatGPT for diverse learning activities	4.17	.745
**Social Influence (SI)**		
My peers suggest me to use ChatGPT for learning and research purposes	4.18	1.066
My teachers encourage the use of ChatGPT for assignments and research activities	3.17	1.412
The use of ChatGPT for learning purposes and assignments is increasingly popular among students in my class	4.07	1.114
**Facilitating Condition (FC)**		
I have the requisite technologies (smartphone, laptop, internet) to utilize ChatGPT for my studies	3.19	1.571
My university offers assistance and resources for utilizing ChatGPT in my studies	2.53	1.006
My university has established explicit standards for the ethical usage of ChatGPT in assignments and research	2.14	1.039
I have financial support for using the Premium edition of ChatGPT	2.39	1.261
**Behavioral Intention (BI)**		
I plan to keep using ChatGPT for my academic pursuits and research activities	3.49	1.035
I plan to increase my use of ChatGPT for future assignments and projects	3.41	1.116
**Actual Use (AU)**		
I regularly use ChatGPT to finish my projects and assignments	3.28	1.171
I frequently utilize ChatGPT to study for my courses and get ready for tests	3.75	.808
I frequently consider the possibility of plagiarism and moral dilemmas when utilizing ChatGPT for research and assignments	4.05	1.170
I frequently verify the information supplied by ChatGPT to make sure it is accurate for the assignments or research	3.68	1.104
I regularly use ChatGPT to prepare Power Point Presentation for academic purposes	4.07	1.078
I actively search for advice or tutorials to help me use ChatGPT more effectively for my academic work	3.89	.890
I try not to rely too much on ChatGPT to help me be creative with the assignments and research tasks	3.16	.959
I use ChatGPT to have language support while writing assignments	3.84	1.090

ChatGPT led to increased efficiency for students and better comprehension of the course material as students reported. It was beneficial with more complex subjects (M=4.47, SD=0.625) and ideas for project generation (M=4.44, SD=0.769). Furthermore, ChatGPT quickly and accurately helped with tasks (M=4.47, SD=0.729). Students often used it to complete assignments (M=4.31, SD=0.591) and prepare presentations (M=4.07, SD=1.078). Moreover, students reported ease of use when integrating ChatGPT into their academic activities, highlighting the low effort to use ChatGPT effectively (M=4.15, SD=1.051). They deemed themselves reasonably competent in using ChatGPT (M=4.17, SD=0.745) and frequently sought advice and tutorials from ChatGPT (M=3.89, SD=0.890). Moreover, Peer support for the use of ChatGPT was widespread (M=4.18, SD=1.066), but institutional support was perceived as insufficient. Clear ethical use standards (M=2.14, SD=1.039) and IT assistance provided by the universities (M=2.53, SD=1.006) received noticeably low ratings. Although indicating a sustained uptake of ChatGPT in academic settings, students expressed moderate to high intentions for continuous use (M = 3.49, SD = 1.035) and more future utilization (M = 3.41, SD = 1.116), they were cautious about overreliance (M=3.16, SD=0.959) and still focused on creativity and academic integrity.

Table 5 shows the descriptive statistics according to six constructs.

**Table 5.** Descriptive statistics according to six constructs.

		N	Mean	Std. Deviation	95% Confidence Interval for Mean	
					Lower bound	Upper bound
PE	Public University Student	213	4.3587	.51155	4.2896	4.4278
	Private University Student	171	4.3287	.57461	4.2419	4.4154
	Public University Teacher	202	3.3485	.51497	3.2771	3.4200
	Private University Teacher	179	3.2715	.56392	3.1883	3.3547
	Total	765	3.8308	.74692	3.7778	3.8839
EE	Public University Student	213	4.1408	.62815	4.0560	4.2257
	Private University Student	171	4.1550	.67799	4.0526	4.2573
	Public University Teacher	202	3.1485	.62859	3.0613	3.2357
	Private University Teacher	179	3.1983	.63305	3.1050	3.2917
	Total	765	3.6614	.80467	3.6043	3.7185
SI	Public University Student	213	3.7762	.64873	3.6886	3.8638
	Private University Student	171	3.8499	.63674	3.7538	3.9460
	Public University Teacher	202	2.9373	.68096	2.8428	3.0318
	Private University Teacher	179	2.9441	.66337	2.8463	3.0420
	Total	765	3.3765	.78805	3.3205	3.4324
FC	Public University Student	213	2.5681	.78462	2.4621	2.6741
	Private University Student	171	2.5512	.82956	2.4259	2.6764
	Public University Teacher	202	2.6609	.70542	2.5630	2.7588
	Private University Teacher	179	2.6969	.71056	2.5921	2.8017
	Total	765	2.6190	.75904	2.5651	2.6728
BI	Public University Student	213	3.4366	.76137	3.3338	3.5395
	Private University Student	171	3.4737	.71180	3.3662	3.5811
	Public University Teacher	202	3.6807	.79380	3.5706	3.7908
	Private University Teacher	179	3.6089	.83675	3.4855	3.7324
	Total	765	3.5497	.78276	3.4941	3.6052
AU	Public University Student	213	3.7048	.61412	3.6219	3.7878
	Private University Student	171	3.7295	.58735	3.6409	3.8182
	Public University Teacher	202	3.6535	.59873	3.5704	3.7365
	Private University Teacher	179	3.6285	.63149	3.5353	3.7216
	Total	765	3.6789	.60841	3.6357	3.7221

The study uncovered distinct patterns among the six variables (PE, EE, SI, FC, BI, AU) highlighting differences in perceptions between the students and the teachers of different public and private universities. In terms of performance expectancy (PE), students at public universities had the highest mean (M=4.36, SD=0.58) for the usefulness of the system for improving performance. Students enrolled in private universities showed a mean score of 4.20 (SD=0.62). Public and private university teachers scored significantly less than the overall score (public university teachers: M=3.72, SD=0.64; private university teachers: M=3.60, SD=0.68). Furthermore, the effort expectancy (EE) trends mirrored those for Performance Expectancy (PE). Public university students obtained the highest (M=4.28, SD=0.57), followed by private university students (M=4.15, SD=0.59). The average score of the public universities teachers EE was 3.68 (SD=0.65), while the lowest average score, 3.55 (SD=0.70), belongs to the private universities teachers. On the other hand, students from a private university showed the maximum mean score (M=3.85, SD=0.64) with the most significant perception of societal pressure to use the system, while public university students had a mean score of 3.72 (SD=0.63). Teachers scored lower on this construct— public university teachers scoring an average of 3.25 (SD=0.60) and private university teachers scoring an average of 3.18 (SD=0.65).

For the facilitating conditions (FC), scores were relatively low across all groups, reflecting a consensus on a lack of support/resources. The students from public universities scored slightly higher (M=3.12, SD=0.75) than those from private university students (M=3.05, SD=0.72). Teachers showed the lowest ratings: teachers in public universities (M=2.95, SD=0.68) and private universities (M=2.90, SD=0.70). However, unlike other constructs, teachers had a higher behavioral intention (BI) than students. The highest mean score (M=4.10, SD=0.65) was identified for public university teachers, followed by private university teachers (M=4.00, SD=0.68). Still, public university students scored an average of 3.88 (SD=0.66), and private university students scored an average of 3.80 (SD=0.68). Lastly, regarding actual use (AU), having little variability, all groups were impressively consistent in how they scored. Public university teachers averaged 3.75 (SD=0.58), slightly ahead of private university teachers (M=3.70, SD=0.60). Students showed similar relative scores, with average scores of public university students of 3.68 (SD=0.63) and private university students of 3.65 (SD=0.64).

**Table 6.** One way ANOVA statistics.

ANOVA						
		Sum of Squares	df	Mean Square	F	Sig.
PE	Between Groups	204.717	3	68.239	234.430	.000
	Within Groups	221.515	761	.291		
	Total	426.232	764			
EE	Between Groups	182.140	3	60.713	147.827	.000
	Within Groups	312.547	761	.411		
	Total	494.687	764			
SI	Between Groups	144.782	3	48.261	111.399	.000
	Within Groups	329.683	761	.433		
	Total	474.465	764			
FC	Between Groups	2.781	3	.927	1.613	.185
	Within Groups	437.395	761	.575		
	Total	440.175	764			
BI	Between Groups	7.806	3	2.602	4.302	.005
	Within Groups	460.306	761	.605		
	Total	468.112	764			
AU	Between Groups	1.167	3	.389	1.051	.369
	Within Groups	281.640	761	.370		
	Total	282.807	764			

The one-way ANOVA indicates that the differences between groups are significant for the constructs of Performance Expectancy (PE), Effort Expectancy (EE), and Social Influence (SI) (p <. 001 for each. PE between-group data showed substantial variation (Sum of Squares = 204.717, F(3, 761) = 234.430, p <. 001), indicating substantial group-level differences. EE also showed significant differences (Sum of Squares = 182.140, F(3, 761) = 147.827, p <. 001) and SI (Sum of Squares = 144.782, F (3, 761) = 111.399, p <. 001). Behavioral Intention (BI) was significantly changed, but less strong effect (Sum of Squares = 7.806, F(3, 761) = 4.302, p =. 005). However, no statistically significant differences were found for Facilitating Conditions (FC) (F(3, 761) = 1.613, p =. 185) or Actual Use (AU) (F(3, 761) = 1.051, p =. 369), indicating that attitudes for these categories were consistent across groups. The results deliver a unique overview of multifaceted heterogeneity in users' attitudes and intentions across multiple cohorts and common challenges in favoring conditions and actual usage behavior, especially regarding perceived usefulness, ease of use, and social influence.

Tukey's HSD post hoc analysis showed significant group-level differences across constructs. Performance Expectancy (PE) and Effort Expectancy (EE): Students in both public and private universities scored higher than teachers (p <. 001), where the highest scorers were the students of public universities. Students outperformed teachers (p <. 001) based on Social Influence (SI), whereas private university students performed the best. By Behavioral Intention (BI): the teachers outscored students, and public university teachers outscored private university teachers. However, the difference was not statistically significant. These findings may suggest a greater propensity for teachers to apply the system and a more excellent perception amongst students of its utility and use.

## Findings from Qualitative analysis

**Figure 2.**
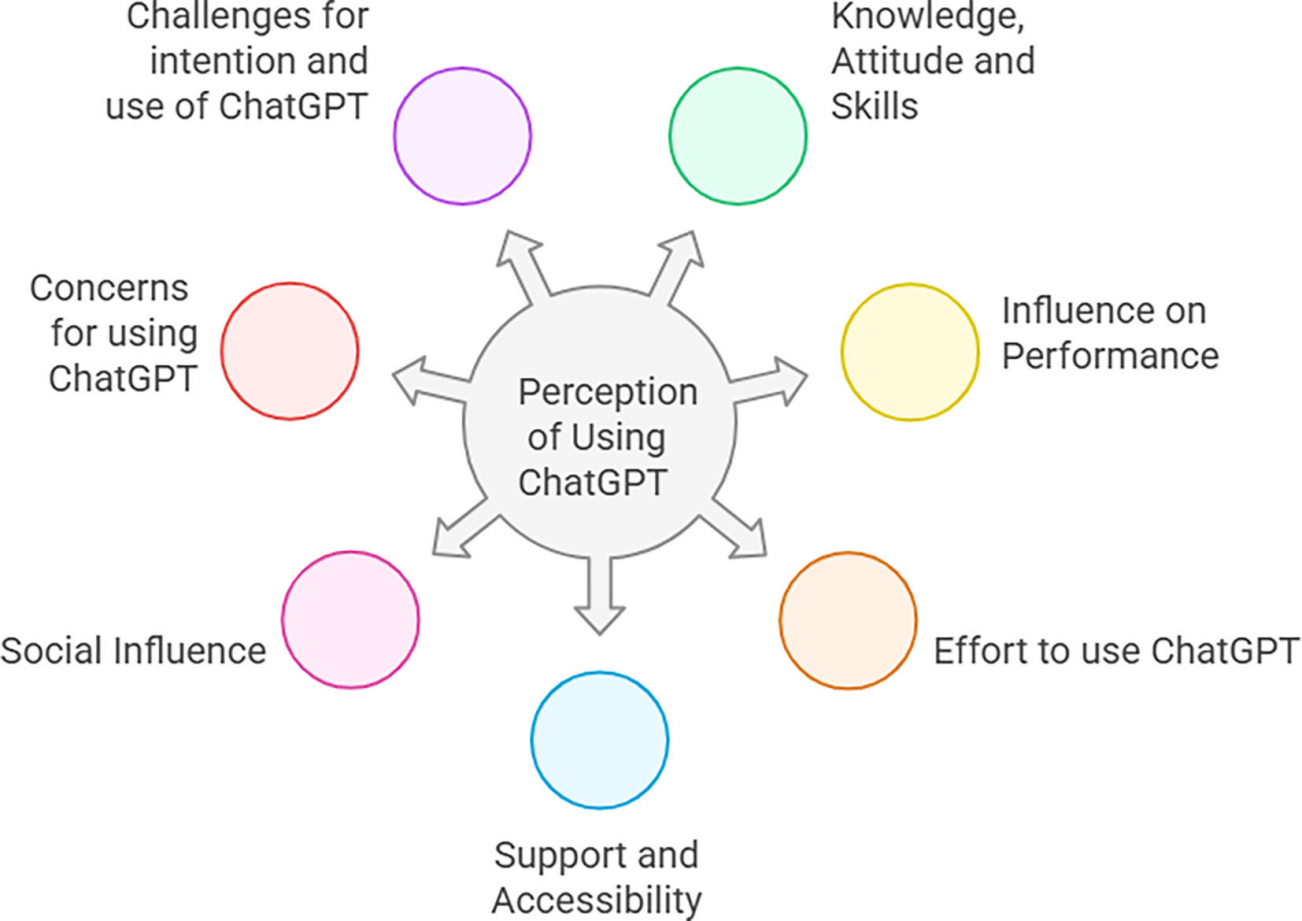
Comprehensive framework from thematic analysis. This figure illustrates a comprehensive framework of this study, which emerged from the thematic analysis of qualitative data from 16 teachers and 16 students in in-depth interviews. It highlights seven key themes, including knowledge, attitude, and skills; influence on performance; effort to use ChatGPT; support and Accessibility; Social Influence; concerns for using ChatGPT; and challenges for intention and use of ChatGPT.

The following figure ( Figure 2) demonstrates a comprehensive framework developed from thematic analysis of qualitative data.

### Knowledge, attitude and skill of using ChatGPT

For students and teachers in Bangladesh, using ChatGPT is an unconventional idea. For both public and private university teachers claim that they need to become more acquainted with ChatGPT as their proficiency in using ChatGPT for teaching is average. Many university students, both public and private, have the necessary knowledge and abilities to make successful use of ChatGPT. The majority of them claim to be sufficiently knowledgeable about ChatGPT. These involve interacting with artificial intelligence as well as navigating and using ChatGPT. With their ability to command and instruct, they have the necessary skills and knowledge to extract accurate data from AI efficiently. Most of them used YouTube tutorial videos to learn how to use ChatGPT. One public university student said,

“I am proficient in utilizing ChatGPT without any difficulty. I employed ChatGPT without undergoing any training. I acquired proficiency in using new programs using YouTube. I utilize it as a means of assistance when I encounter difficulties.”

However, ChatGPT is highly regarded by both students and teachers at private universities. Private university students and teachers have shown great interest in and excitement about using ChatGPT. Using ChatGPT, users can quickly receive answers to their questions and verify the accuracy of their information. Some teachers in public universities, on the other hand, have a negative outlook. Some teachers have reported negative consequences for teachers and students due to using ChatGPT. Some teachers believe using ChatGPT in the classroom can jeopardize current teaching strategies and possibly reduce teachers' efficacy. Furthermore, several teachers contend that ChatGPT impedes students' intellectual growth. A public university teacher said,

“ChatGPT is fostering a sense of laziness among our adolescent learners. Currently, students are quickly accessing information through ChatGPT, diminishing their reliance on cognitive abilities. Consequently, students' creativity and mental abilities are being impaired.”

### Influence on performance

ChatGPT is viewed favorably by faculty members at private universities when it comes to creating course materials. According to several teachers, ChatGPT is a tool that helps them better comprehend any subject and improves their knowledge of the material. Teachers at public universities point out that ChatGPT's ability to offer thorough information on a range of topics is limited. Though the teachers acknowledged that relying solely on ChatGPT for developing classroom presentations is inappropriate, they foud it convenient to having a comprehensive review of the topic. Both teachers in public and private universities acknowledge that ChatGPT enhances their capacity to address students' inquiries, elucidate concepts, generate stimulating discussion topics, perform language translations, and generate personalized learning materials such as quizzes and flashcards. It guides the most effective teaching strategies or tactics for a specific subject. Moreover, it facilitates designing lessons and provides comprehensive expertise in several subjects, positively impacting their professional competence. A teacher at a private university remarked,

“Previously, I needed a comprehensive grasp of several topics. However, I now endeavor to enhance my knowledge by utilizing ChatGPT, which has resulted in improved academic achievement. I have learned the appropriate techniques to employ for different types of content.”

Through ChatGPT, faculty members at public and private universities can easily perform research activities such as idea generation, setting research objectives, developing theoretical frameworks, evaluating literature, and developing techniques. However, most teachers are hesitant to use ChatGPT in their domain because of the inaccurate and deceptive information it produces. The links or sources that ChatGPT offers are not trustworthy. Several academics argue that ChatGPT's citations to DOI (Digital Object Identifier) are inaccurate. On the other hand, both students at public and private universities concur that it enhances their academic outcomes. With ChatGPT, users may easily comprehend the subject matter and obtain suggestions for assignments or presentations, directly influencing their academic achievements. It enhances students' learning efficiency, hence facilitating the attainment of their learning goals. In addition, numerous students at public universities assert that they can utilize it to disseminate content through different approaches. Currently, students in the classroom exhibit heightened receptiveness and increased engagement compared to previous instances. A student of a public university claimed,

“Utilizing ChatGPT has dramatically enhanced our learning efficacy. Now, it is straightforward to acquire a comprehension of the predetermined syllabus. Consequently, our active engagement in the classroom learning process has intensified.”

Besides, both private and public university students concur that it facilitates their study and research. They increasingly rely on ChatGPT to obtain answers to a wide range of issues. They acquire a diverse range of knowledge from it. It facilitates the development of their essays, reports, and compositions for monographs. They get advantages from it while creating presentation slides and collaborating in groups. They also derive benefits from it for their thesis. A student of a private university said,

“The little time given for assignments poses a significant challenge in creating a well-crafted task within a tight timeframe. With the assistance of ChatGPT, I can now efficiently generate well-informed and suitable assignments in a short period.”

Teachers and students at public and private universities point out the different kinds of assistance and guidance that are accessible when using ChatGPT. They spend the same time looking through numerous search engines to read stuff. It also helps them overcome the challenges presented by the English language, simplify complex ideas, and create educational materials. It assists people in improving their subject matter, pedagogical, and technological proficiency. Teachers and students at both public and private universities have shown their support for ChatGPT. ChatGPT is reportedly expanding its knowledge base and improving its research and analytical skills. Several students highlight that ChatGPT's constant availability allows them to seek assistance beyond regular class hours. Students also noted that ChatGPT has the potential to assist them in addressing a problem by considering multiple perspectives, thus enhancing their capacity for critical and creative thinking from different angles. A student of a private university said,

“ChatGPT has expanded the scope of my learning. The processing time for every task is minimal. Each time I inquire, I promptly receive a highly rational explanation, enhancing my cognitive abilities.”

### Effort to use ChatGPT

All participants assert that the ChatGPT interface is user-friendly. Consequently, all individuals can use ChatGPT without encountering significant challenges. They can function and traverse effortlessly as a result of a straightforward interface. All participants acknowledge that ChatGPT provides prompt responses. It uses simple and easily understandable language. It endeavors to illustrate any idea using examples and in a straightforward manner. A student from the public university said,

“When I used ChatGPT for the first time , I thought it might be complex. As I am not handy to technology, but I found simple steps to use it. I just need to provide specific command to get sufficient answer.”

However, teachers of both public and private universities considered the interface of ChatGPT user friendly . Though it is easy to operate, they are concerned for using it in academic research purpose due to lack of specific guideline to use it in the research. One of the private university teachers mentioned,

“It is good to use ChatGPT to get a basic idea about research. It even helps guide the theoretical framework. However, my concern is how I will use it in the study, as I do not know whether specific guideline prevails or not to use it. As a result, I have not tried to use it in research. I use it to get some basic ideas.”

### Support and accessibility

Participants opine they use multiple electronic devices and operating systems (smartphone, tablet, laptop, desktop computer) to access ChatGPT. However, they require strong networks and speedy internet connections. Sometimes, network problems can stop them from using ChatGPT. Everyone agrees that ChatGPT also works in other languages, but its answers in English are always better. It fails to give correct answers in Bangla in some cases and misinterprets commands sent in Bangla at times. In addition, study participants asserted that they could not use the ChatGPT Plus subscription edition. All the participant did not get any institutional support to use ChatGPT Given the budgetary constraints, many teachers and students in Bangladesh need help to afford the expensive fees associated with premium usage—consequently, users of the complimentary version express dissatisfaction with the few resources provided by ChatGPT. A private university student said,

“I use the accessible version of ChatGPT. I cannot buy the premium version as it is costly. Sometimes, I do not get answers from ChatGPT about my queries.”

A teacher of public university said,

“I think to cope with present era, we cannot ignore AI like ChatGPT. We need to get access to be updated ourselves by using this tools but our financial barrier impedes us to use ChatGPT-4. Universities can support in this sector.”

### Social influence

Most of the students from public and private universities admitted that they were inspired to use ChatGPT by their peers. Peers who are good at using ICT, they mainly influence other students to use ChatGPT for assignment, presentation slide, and various queries. A private university student said,

“When I struggled to prepare my assignment, my classmate suggested using ChatGPT. For his inspiration, I signed up for the ChatGPT free version, and he was waiting for me. I got everything that I needed. I embraced my friend for his suggestion.”

Few students get inspiration from teachers to get basic ideas of different complex term, but teachers are not influenced by their colleagues. Most of the teachers of public and private universities admitted that they were not influenced by their colleague to use ChatGPT or other AI tools.

A public university teacher said,

“We are swamped taking classes as it is a teaching university. We do not have time to discuss innovations or things like that. If we meet, we chitchat to relax. Hardly academic discussion we do.”

### Concern for using of ChatGPT

Most faculty members know its reliability, indicating that they possess an elementary awareness of its advantages and disadvantages. Teachers have noted that it sometimes provides inaccurate information. The system autonomously generates information; however, its information is unreliable. It generates random and imprecise points of reference. Occasionally, it needs to more comprehend the context of a query, leading to responses that appear irrelevant or unrelated. Furthermore, it generates stuff that is hypothetical or fictional. In addition, several educators express dissatisfaction with its practice of distributing identical content to multiple users, which diminishes their confidence in its effectiveness. In this regard, a teacher of a public university stated,

“I have reservations about ChatGPT due to its tendency to disseminate inaccurate content and its need for more transparency on the sources of information. I solely utilize it for grasping the fundamentals, as I need clarification regarding the reliability of the content.”

However, all students admit that they use ChatGPT without fully understanding its workings. They never doubt the precision of ChatGPT. As digital technologies become increasingly prevalent in classrooms and other learning environments, students often need to pay more attention to their limitations and potential for error. One public university student mentioned,

“I am not worried about the reliability of the AI information because it makes my life more enjoyable. I finished my assignments very quickly with the help of ChatGPT.”

Issues regarding the ethical implications of ChatGPT are expressed by all university teachers, whether from private or public universities. Using material that requires author acknowledgment might be difficult for teachers due to ethical constraints. Several academics statethat there is no standard procedure for keeping track of data produced via ChatGPT. Therefore, more extended data retention periods give rise to privacy and data security issues. In addition, teachers at both public and private universities note that students require more excellent education regarding the moral implications of ChatGPT. While additional plagiarism detection methods are needed and their use is limited, teachers cannot evaluate the authenticity of their students' assignments, reports, and monographs. As a result, using ChatGPT requires adherence to institutional rules and policies. Teachers at private and public universities acknowledge the potential disadvantages of ChatGPT in educational settings and take steps to deal with them. They express concerns over excessive dependence on ChatGPT. They are concerned that relying on it too often may hinder their capacity to learn autonomously and solve difficulties. Teachers at private universities complain that ChatGPT is fostering complacency among them. Teachers employ ChatGPT to confine their expertise inside a specific domain, unlike their predecessors, who would amass their knowledge by perusing several books, publications, and articles. Excessive reliance on ChatGPT may undermine teachers' capacity for critical thinking and independent inquiry. In this regard, a teacher at a private institution stated that,

“ChatGPT has fostered our reliance on technology. Previously, we relied on diverse sources to acquire knowledge, while now we can promptly get information. Its detrimental impact outweighs its beneficial effects. We are diminishing our cognitive abilities.”

Teachers are reluctant to exclusively utilize technology for their advantage due to concerns for their students. Most university faculty members acknowledge the potential danger of excessive reliance on technology as students grow increasingly intertwined with artificial intelligence. Consequently, students' capacity for critical thinking and problem-solving is reduced. The utilization of ChatGPT by students is hindering their potential to cultivate autonomous thinking and originality, as well as their aptitude to engage with people, communicate proficiently, and foster commendable reading and writing practices. One of them remarked,

“Previously, students acquired knowledge by engaging in peer discussions. Nevertheless, their current utilization of ChatGPT for information retrieval impedes their consciousness development.”

However, university students and teachers concur that educational establishments can mitigate the adverse impacts by fostering critical thinking, digital literacy, well-rounded integration, and ethical considerations. Furthermore, they believe continuous assessment and adjustment are vital to ensure that AI improves rather than diminishes these tools. It is necessary to develop and enforce specific policies for utilizing ChatGPT in educational and research endeavors to mitigate potential adverse consequences arising from its use over an extended period **.**

### Challenges for intention and use of ChatGPT

The majority of participants encounter diverse challenges when using ChatGPT. Several teachers emphasize the limitations of ChatGPT, such as its tendency to present biased or inaccurate material, its need for more subject-specific expertise, and its tendency to provide superficial or shallow information. Teachers need help to place confidence in the content produced by ChatGPT due to its failure to attribute credit to the original authors of the information which decrease their intention to use ChatGPT in research purpose. Evaluating the authenticity and novelty of assignments provided by students through ChatGPT also poses difficulties for teachers. Academic integrity is compromised when students exploit ChatGPT to generate plagiarised content for their assignments and examinations. Teachers face challenges in fostering critical and profound engagement among their students, as they can quickly obtain answers using ChatGPT. Considering the current prevalence of student attachment to ChatGPT, all teachers unanimously agree that ChatGPT poses some challenges. In this regard, public university teachers stated,

“Within our educational system, a significant proportion of the students enroll in higher education institutions to acquire a degree, which serves as a means to ensure future work opportunities. They continuously assess their efficiency in earning degrees. These students are fortunate to have access to ChatGPT but they are reluctant to attend classes.”

While utilizing ChatGPT, students face many challenges in conjunction with lecturers. Students also met the language intricacy of ChatGPT, which hindered their comprehension of the information it conveyed. Most students held unfavorable views on the exorbitant cost of ChatGPT's premium edition, as it was outside their financial means. ChatGPT users may encounter technical difficulties such as system crashes, malfunctions, network challenges, or internet connectivity problems.

## Discussion

Bangladesh, as a developing nation, is undergoing through a transformation since it has been trying to integrate technology into several sectors, including education. The swift progress in Natural Language Processing technology has allowed educational institutions to consider incorporating innovative ways of instruction and learning. Teachers and students in the tertiary educational institutions at Bangladoesh use ChatGPT for their academic needs. According to this study, students of both public and private universities use ChatGPT as a highly immersive platform, demonstrating a high-performance expectation across the platform. This finding aligns with the research done by Limna et al. (2023), which found that students think ChatGPT helps them learn complex subjects. However, performance expectancy scores were lower for teachers than students at both public and private universities. This discrepancy can result from teachers' cautious views regarding ChatGPT's effects on learning environments. According to study by Kasneci et al. (2023), teachers are more suspicious about the practical usage of ChatGPT in teaching and research, even when students adapt it to some degree due to possible benefits. According to the effort expectancy results for both student groups of this study, ChatGPT was user-friendly. This is in line with Ollivier et al. (2023), who highlight how easy it may be for students to use AI tools. Both public and private university teachers’ have moderate level effort expectancy scores that also reflected in Sok and Heng (2023) study while they resonates with the need for professional development that can boost confidence and ease of use of AI.

Social influence is an important factor for ChatGPT usage. The findings show a moderate social influence on students' use of ChatGPT, indicating that peer support plays a significant role in students' engagement with AI tools. These findings support earlier research suggesting that classroom social dynamics significantly influence students' use of technology ( Winstone & Boud, 2022). On the other hand, both teacher groups scored lower on social influence, indicating that their institutions and peers don't encourage them to use ChatGPT. Dawa et al. (2023) emphasize that positive feedback from colleagues increases teachers' ChatGPT usage and encourages them to adopt it. ChatGPT usage also depends on some facilitating conditions. All teachers and student groups have identified the availability of technology resources and the institution's support for using ChatGPT in an educational setting as barriers and poor facilitating conditions. This is consistent with studies by Limna et al. (2023) that highlight the need to have sufficient infrastructure and support mechanisms to ensure successful technology integration. These results also highlight a critical area for educational institutions to address since the potential benefits of AI technologies for teaching and learning might be seriously hampered by a lack of supportive environments.

Teachers exhibited a stronger behavioral intention to use ChatGPT than their students, although students in both groups expressed a modest intention to use ChatGPT in the future. This means teachers are more committed to employing these tools in their practices, even when students desire to use them. This result is consistent with studies that indicate teachers are beginning to recognize the benefits of using AI tools to improve their teaching ( Ollivier et al., 2023). However, it also emphasizes how crucial ongoing assistance and training are to guarantee this commitment. According to the actual use scores of this study, both groups of students frequently utilize ChatGPT for various academic assignments, which highlight how actively they engage with AI technologies in their studies. This is also consistent with research by Limna et al. (2023) that shows students want to use technology to improve their educational experiences. Though their ratings were lower than those of students, teacher usage rates were also quite high, suggesting impediments to integration into teaching roles that are in line with Sok and Heng's (2023) findings.

In addition to the various benefits of employing ChatGPT for academic purposes, a few teachers agree that the utilization of ChatGPT carries specific adverse ramifications for both teachers and students. Excessive comfort in working environments may hinder pupils' intellectual maturation by suppressing their need to engage in critical thinking. Moreover, Bai et al. (2023) found that an overabundance of ChatGPT usage leads to a diminished ability to engage in critical thinking. ChatGPT has been identified as the cause of decreased memory recall. Furthermore, it might also hinder the professional growth of educators. Teachers at private universities utilize ChatGPT to enhance their lecture preparation and expand their expertise in various subjects. Conversely, teachers at public universities believe that ChatGPT's knowledge base is limited and primarily receptive rather than comprehensive. Garg et al. (2023) and Spennemann (2023) claim that though ChatGPT is receptive to information, its capacity to provide extensive knowledge on certain subjects is very constrained.

According to Castillo et al. (2023), most students utilize ChatGPT due to its rapidity and precision, which benefit their learning process. Being a user from Bangladesh, individuals occasionally need some help due to the limitations of ChatGPT in providing proper responses in the Bangla language. It frequently misinterprets orders given in Bangla, which aligns with the research conducted by Kolar and Kumar (2023) on Hindi, Telugu, and Kannada. Furthermore, the requirement of a ChatGPT Plus subscription presents an additional obstacle to utilizing ChatGPT. Bangladesh is classified as a lower-middle-income country. In Bangladesh, there are lots of resource constraints to use online for teaching-learning like network issues, internet connectivity and speed, financial barriers ( Rahman et al., 2023). Hernandez (2019) posits that the financial constraints faced by hinder its ability to adapt technology in several sectors where it is required effectively. Similarly, most teachers and students in Bangladesh need help to afford the expensive fees associated with premium usage, primarily due to budgetary constraints. Consequently, consumers encounter numerous problems when using the free edition of ChatGPT.

ChatGPT has been found to exhibit inaccuracies or misinformation. Since it can autonomously provide information that could be more reliable, furthermore, it occasionally shows a deficiency in comprehending the context of a query, leading to the provision of irrelevant responses. Moreover, it generates content that is speculative or fictional. Nevertheless, despite all the constraints, the students use ChatGPT without considering its drawbacks. As students get more familiar with digital tools, they may need to pay more attention to the increasing prevalence of faults in academic procedures. However, teachers have limitations in assessing the authenticity of their students' assignments, reports, and monographs because of the absence of plagiarism detection systems and their limited accessibility. Consequently, adhering to institutional regulations and guidelines is necessary to utilize ChatGPT. Limna et al. (2023) have highlighted the necessity of establishing an institutional policy to mitigate the disadvantages associated with using ChatGPT.

Teachers possess knowledge of the potential limitations of ChatGPT when used in educational settings. Despite their continual usage, they have concerns that it may hinder their capacity to learn autonomously and resolve issues. Contemporary educators utilize ChatGPT to confine their expertise to a particular domain, unlike their predecessors, who would amass their information by perusing several books, publications, and articles. The overreliance on ChatGPT may undermine teachers' capacity for critical thinking and independent inquiry. Teachers express concerns about their students' overreliance on technology and artificial intelligence. Consequently, students' cognitive skills, such as critical thinking and problem-solving, may be impaired. Nevertheless, there is a consensus among both educators and learners that educational establishments can mitigate the adverse consequences by fostering critical thinking, digital literacy, well-balanced incorporation of AI technology, and ethical considerations. Explicit policies must be formulated and executed to utilize ChatGPT in educational and research endeavors.

### Implication

The results elucidate the potential, utility and implications of the adaption of ChatGPT in higher education of Bangladesh and other similar contexts. The research also offers recommendations for maximizing ChatGPT's benefits, addressing challenges, and integrating it successfully. Understanding the views of social science faculty teachers and students at the private and public universities in Bangladesh regarding use of ChatGPT are important to take informed decisions, develop sound policies, and establish AI technologies in the education system. This study aims to provide a more engaging, innovative and efficient teaching- learning environment for Bangladesh's higher education system from the viewpoint of diverse aspects. The findings of this study will also be relevant for developing and underdeveloped countries with socio-economic and cultural contexts similar to Bangladesh's, contributing to their higher education systems.

### Limitation

This study only focused on the teachers and students of social science faculty from multiple universities in Bangladesh, so the findings may not reflect the holistic perspective of ChatGPT usage among all demographic user categories. Focusing on a single set of users limits the generalizability of the findings to a larger population because users from other areas of interest or geography may have completely different experiences and perceptions.

## Conclusion

This study endeavors to investigate the perception and use of ChatGPT by social science faculty teachers and students at public and private universities in Bangladesh for instructional purposes. This study reveals several noteworthy concerns that require further contemplation and examination. The study's findings suggest that while the majority of students in both public and private universities demonstrate proficiency in utilizing ChatGPT, there exists a notable discrepancy in the expertise levels of teachers. Moreover, teachers hold divergent perspectives on using ChatGPT, with some displaying enthusiasm while others expressing skepticism. Nevertheless, students at both types of universities view ChatGPT favorably for a range of educational endeavors and learning prospects, such as improved knowledge and research capabilities. Students from both private and public universities affirm that utilizing ChatGPT improves their learning outcomes and positively impacts their academic achievement. ChatGPT is also helpful for teachers. It enhances their teaching efficacy by assisting in class planning and providing comprehensive expertise on the subject matter.

Nevertheless, owing to the exorbitant cost of ChatGPT premium, users resort to utilizing the free version that offers restricted functionalities. Robust networks and high-speed internet are essential for enhancing the accessibility of ChatGPT. Furthermore, the efficiency of the English language and the limitations of other languages pose a significant obstacle to its effective utilization. This study also investigates the apprehension regarding the dependability of information offered by ChatGPT due to potential misinterpretation of queries. Furthermore, the ethical applications need to be more credible due to the absence of citations. While teachers express concerns over the ethical implications of utilizing ChatGPT and the potential for excessive reliance on it, students from both public and private universities still need to be made aware of these difficulties. After carefully examining and establishing a significant relationship between these findings and the broader implications of the research, it is clear that it is essential to eliminate barriers, including addressing ethical concerns, to ensure equitable access to ChatGPT and harness its potential to enhance the quality of teaching, learning, and research in higher education. Future research should explore novel approaches to overcome these challenges and maximize the benefits of ChatGPT in various educational settings.

## Ethical approval statement

This study adhered to the ethical guidelines in the Declaration of Helsinki and received approval from the Institute of Education and Research Human Research Ethics Committee (IERHREC) with approval number 2023/01 on 10/02/2023. The protocol for the study was reviewed to ensure its adherence to ethical norms governing research involving humans. This review encompassed the protocol's aims, methodology, and informed consent processes. All individuals who took part in this study provided their consent before participating, and their confidentiality and privacy rights were protected during every stage of the research procedure. All potential risks to the participants were minimized.

## Informed consent

Researchers informed the study objectives and privacy conservation issue to the participants and provided written consent letter to the participants. After getting consent from the participants, researchers collected data.

## Authors contribution statement

Conceptualization and planning by 1 ^st^ author, tools development by 2 ^nd^ author, data collection and data analysis by 3 ^rd^ and 4 ^th^ author; writing introduction, literature review, and implication by 1 ^st^ author, theoretical framework and methodology by 2 ^nd^ author, discussion by 3 ^rd^ author, conclusion by 4 ^th^ author; supervision, review and editing by 1 ^st^ and 2 ^nd^ author. All authors read the manuscript thoroughly and agreed to submit and publish in this journal.

## Data Availability

Figshare: *Teachers’ and students’ use of ChatGPT at Social science faculty in the public and private Universities of Bangladesh*, https://doi.org/10.6084/m9.figshare.28351223 ( Rahman *et al*., 2025a) This study dataset entitled consists of the following three data: Dataset1—This data set consists of 402 teacher survey responses from social science faculty of public and private universities about their perceptions and practices of using ChatGPT. Dataset2—This data set consists of 440 student survey responses from social science faculty of public and private universities about their perceptions and practices of using ChatGPT. Dataset3—This dataset consists of qualitative data from 16 teachers and 16 students in-depth interviews. These data are available under the license CC BY 4.0, which allows unrestricted use and reproduction. To ensure confidentiality and privacy, all data sets have been attached after hiding participants' identities. **Figshare:** *Supplementary file for ChatGPT use of social science faculty teachers and students.* 10.6084/m9.figshare.28368161 ( Rahman et al., 2025b) This supplementary file consist of three data file as an extended data: Survey questionnaire: Two survey questionnaires used for teachers and students of the social science faculty of both public and private universities of Bangladesh in this study. These data are available under the license CC BY 4.0, which allows unrestricted use and reproduction. To ensure confidentiality and privacy, all data from supplementary file have been attached after hiding participants' identities.

## Software availability statement

IBM SPSS: IBM SPSS is an data analysis software usually use for social science research. It is available from IBM Corp. (2021). https://www.ibm.com/support/pages/downloading-ibm-spss-statistics-27

JASP: JASP is an open-source statistical software for data analysis which can perform similar functionalities like SPSS. It is freely available at https://jasp-stats.org
