# Teachers’ and students’ use of ChatGPT at Social science faculty in the public and private Universities of Bangladesh

**DOI:** 10.12688/f1000research.161351.2

**Published:** 2025-09-05

**Authors:** Arifur Rahman, Md Khairul Islam, Abdullah Al-Mamun, Md Shahidul Islam

**Affiliations:** 1 Institute of Education and Research, University of Dhaka, University of Dhaka, Dhaka, Dhaka, 1000, Bangladesh; 2 Institute of Education and Research, University of Dhaka, Dhaka, Dhaka, 1000, Bangladesh; 3 Institute of Education and Research, University of Dhaka, Dhaka, Dhaka, 1000, Bangladesh; 4 Jaggo Foundation, Rajshahi, Rajshahi, 6205, Bangladesh

**Keywords:** ChatGPT, teaching-learning, perception, academic integrity, reliability, ethical consideration, higher education

## Abstract

**Background:**

Bangladesh is an emerging country where teachers and students of public and private universities have started using technology in the classrooms. Many teachers and students of social science faculty have an inclination to use ChatGPT for educational and research purposes. This study, centering on this specific context, aims to provide insights into the perception and integration of ChatGPT into the educational practices of an emerging country.

**Methods:**

This study employed a mixed method approach. Quantitative data were collected through questionnaire survey from 402 teachers and 440 students of eight different public and private universities following a stratified sampling approach. A convenience sampling technique was followed with a view to collecting qualitative data through in-depth interviews of 32 participants, comprising 16 teachers and 16 students from both public and private universities.

**Results:**

The study presents that students and teachers both have proficiency, yet a gap in expertise persists. Students perceive ChatGPT as beneficial for better learning outcomes, and teachers find it helpful in preparing for classes and instructional materials. Both teachers and students regard ChatGPT requiring minimal effort. While peer influence drives students to use it, this factor does not influence teachers. However, teachers express stronger behavioral intentions to use it in the future compared to students. Nevertheless, ethical use, reliance, and information accuracy continue to raise concerns. Besides, high cost and language barriers are also listed as reasons for limiting accessibility.

**Conclusion:**

The findings of this study have significant implications for the development of policies, research endeavors, and teaching-learning practices in the higher education sector covering both public and private universities in Bangladesh and other similar contexts.

## Introduction

ChatGPT, an AI technology bearding the GPT (Generative Pre-trained Transformer) architecture, can reasonably improve the teaching and learning process due to its sage conversational capabilities ( Baidoo-Anu & Ansah, 2023). It can provide translation, summary, and question answers, and simultaneously, the generation of texts automates pestering ( Cotton et al., 2023). Unsupervised pre-training and controlled fine-tuning generate responses similar to what a human expert would say, displaying domain knowledge about various topics and answering questions accordingly. Textual data is gathered from various internet sources, including websites, articles, papers, and forums ( Dwivedi et al., 2023). The most remarkable feature of ChatGPT is its ability to generate text. Within just three months of the collective release of ChatGPT, significant numbers of software engineers, authors, academics, teachers, and songwriters used the system to generate written content, academic essays, computer programs, applications, and song lyrics ( Mariani et al., 2023). ChatGPT-4 has continued to be developed, and today, it is used across different applications, including education worldwide. It also helps promoting students’ educational experiences through tailored coaching and support. Implementing this technology has advanced student engagement and collaboration by allowing students to question and debate asynchronously, removing the need to be simultaneously present ( Li & Xing, 2021). ChatGPT can facilitate teachers and students discussing complex ideas at their preferred pace. It also helps with administrative tasks such as responding to commonly asked questions, providing course materials, and managing scheduling or registration tasks. Universities can utilize ChatGPT to improve student engagement by providing continuous support and accelerating administrative processes ( Biswas, 2023).

However, despite these advantages, several critical concerns must be taken under consideration before utilizing ChatGPT. In the context of educational assessment, plagiarism comes as a threat. AI essay-writing systems produce essays based on present parameters or guiding principles which can result in academic forgery by submitting plagiarized write-ups. It counters the key central idea of education, which is to educate and inspire learners, and it could undermine the credibility of academic degrees in the coming years ( Dehouche, 2021). So, the role of ChatGPT in education has sparked and initiated one of the most talked about debates in academia. Therefore, further research is needed to determine ChatGPT adoption in developed and developing countries. An in-depth analysis shows that comparatively, more affluent countries are moving quickly on the chatbots issue and developing ways to leverage it for education. However, developing countries such as Bangladesh need to focus on this. Because technologies do not have borders, understanding the utilization of ChatGPT by a diverse country like Bangladesh could provide a worldwide perspective of the benefits and challenges of adopting and adapting AI technologies in multiple socio-cultural and economic environments. Bangladesh is a developing country where the educational system is being modernized, and technology is being introduced in the classrooms of public and private universities ( Sarker et al., 2019).

Recently, a growing number of teachers and students at tertiary education sector in Bangladesh have been shown interested in using ChatGPT. By focusing on this specific context, the study aims to bring insight into the perception and integration of emerging advanced technology such as ChatGPT into the educational practices of an emerging country. Focusing on Bangladesh as a specific research context, the study aspires to produce meaningful and enlightening knowledge for the worldwide community. In the backdrop of social science faculty at the public and private universities of Bangladesh, where teaching and personalized learning pathways are of great importance, it is essential to address how teachers and students are using ChatGPT. Comparatively, the educational system of Bangladesh has its drawbacks, with large classroom setups, shortage of resources, and potentially low student participation ( Prodhan, 2016). These issues may be mitigated through ChatGPT, which provides personalized support, immediate feedback, and a highly interactive learning environment. Understanding the perception of the key stakeholders’ regarding the use of ChatGPT is crucial to harness this type of advanced technology effectively. Under this circumstance, getting insight into teachers’ and students’ perspectives on employing ChatGPT cannot be neglected because they are an indispensable part of the teaching-learning process.

There is a lack of knowledge about the perceptions and use of ChatGPT among teachers and students in the general settings of social science faculty at public and private universities in Bangladesh. In order to comprehensively evaluate the potential and challenges of implementing ChatGPT in the teaching-learning process in developing countries such as Bangladesh, it is crucial to gain insights into teachers’ and students’ perceptions and usage of ChatGPT. Understanding the perspectives and usage of ChatGPT by teachers and students at social science faculty in developing nations on ChatGPT is essential to ensure its implementation aligns with their specific needs and circumstances. Thus, the aim of this study is to investigate the perceptions and usage of ChatGPT among teachers and students of the social science faculty at public and private universities in Bangladesh, aiming to fill the research gap and contribute to policy-making at universities regarding the use of ChatGPT. Based on this aim, the study addresses the following research question: What are the perceptions and usage of ChatGPT among teachers in the social science faculties of public and private universities in Bangladesh?

## Literature review

Researchers have underscored the significance of understanding the perspectives of stakeholders in order to successfully facilitate the adoption and implementation of AI tools in educational ecology. The application of Artificial Intelligence (AI) in the field of education is an area that elicits controversy and disagreement. According to Rawas (2024), AI systems like ChatGPT have the potential to endanger the privacy of stakeholders, including teachers and students. To demonstrate that ChatGPT is being used as intended and does not have any unintended negative impacts on students or teachers, its use must be transparent and accountable. Moreover, Samala et al. (2025) have mentioned that using ChatGPT raises problems due to specific unfavorable attributes, including technological constraints, ethical dilemmas, plagiarism, deceit, and abuse. In addition, Chassignol et al. (2018) think that these technologies can also adversely impact human interactions, relationships, and the quality of learning. Furthermore, it neglects social engagement and the communal elements of the learning process. Sok and Heng (2024) have raised concerns regarding security, privacy, trust, inaccurate information and an overreliance on technology.

Conversely, numerous studies have focused on the advantages of AI and the positive perceptions of AI held by instructors and pupils. Research conducted by Zawacki-Richter et al. (2019) indicates that teachers generally hold favorable views toward AI and consider it a helpful tool for enhancing educational practices and increasing student engagement. Kaswan et al. (2024) state that AI can offer adaptable feedback, personalized learning opportunities, and intelligent coaching methods. Therefore, students typically hold favorable views toward AI and perceive it as a valuable tool that enriches their educational experiences. They value the ability of AI to offer personalized learning suggestions, immediate feedback, and engaging learning settings. It enhances students’ self-assurance in their aptitude to acquire knowledge and renders the studying process more captivating for them. Vázquez-Parra et al. (2024) have found that university students positively perceived artificial intelligence (AI) as a valuable tool for learning. AI is capable of effectively addressing any inquiries related to topic comprehension.

Numerous AI-based technologies have been developed for educational purposes. Chatbots, an artificial intelligence (AI) tool, offer multiple benefits to education, assisting teachers and students in diverse ways. Sok and Heng (2023) indicate that ChatGPt helps with brainstorming, developing learning assessments, improving pedagogical practices, providing virtual personal tuition, and constructing an outline for an essay or research article. In addition, Chatbots provide tailored learning experiences, support students, improve tutoring and homework assistance, facilitate language acquisition, enable assessment and feedback, enhance student involvement, and aid in research and information retrieval ( Pérez et al., 2020). ChatGPT is gaining popularity in educational environments due to its advanced artificial intelligence capabilities. The GPT series developed by OpenAI, including ChatGPT, utilizes robust and extensive language modeling approaches. As a result of being trained on a wide range of varied texts, the AI model can respond in a manner that resembles human-like behavior and demonstrates a heightened level of comprehension of language ( Dentella et al., 2024).

ChatGPT stands out due to its capacity to enable more sophisticated and contextually relevant conversations. ChatGPT’s remarkable versatility enables it to actively participate in diverse educational endeavors such as tailored learning, student assistance, tutoring, and evaluation. Due to its capacity to comprehend intricate inquiries, deliver precise information, and provide comprehensive arguments, it enhances the efficacy of learning. Kasneci et al. (2023) have found that ChatGPT can aid educators and learners in improving their proficiency in language acquisition, data comprehension, analytical thinking, logical reasoning, and academic research. Although ChatGPT holds significant value in education, it has faced substantial criticism due to its potential to undermine academic integrity. Several issues that require attention include copyright infringement, discrimination, inequity, data privacy and security concerns, and excessive dependence of students or teachers on ChatGPT ( Wu et al., 2024).

Shoufan (2023) conducted a study to determine students’ perceptions of using ChatGPT. The findings demonstrate that students are impressed by the capabilities of ChatGPT and its utility as a tool for studying and working. Users value the humanoid interface of the system for its user-friendly nature and ability to provide well-structured and logically sound responses. Nevertheless, many students contend that the responses provided by ChatGPT lack consistent accuracy. Technology cannot substitute human intelligence, and it is widely accepted that a solid knowledge base is crucial for effectively engaging with it. Firat (2023) suggests that incorporating ChatGPT into education presents numerous opportunities to enhance learning outcomes, tailor instruction, and transform the role of teachers. However, this progress needs to be improved regarding assessment, digital literacy, and ethical considerations. It is essential to maximize the capabilities of ChatGPT entirely by tackling these issues and devising strategies to ensure ethical and fair implementation.

According to a study conducted by Mogavi et al. (2023), the aspects of ChatGPT that are most commonly disputed are creativity and ethics. While ChatGPT is seen as a pioneering advancement by certain initial users, with the potential to boost students’ self-confidence and motivation to learn, there are apprehensions that excessive dependence on the AI system might promote superficial learning habits that could impede students’ critical thinking skills. In another study, Cotton et al. (2023) have presented a series of tactics that institutions can adopt to guarantee the ethical and conscientious utilization of ChatGPT. The strategies encompass implementing standards and processes, offering training and support, and employing various tools to identify and deter cheating.

To comprehensively evaluate the potential and challenges of implementing ChatGPT in developing countries such as Bangladesh, it is crucial to gain insights into the perceptions of teachers and students towards this technology. Gaining insight into the perspectives of teachers and students regarding the assistance provided by ChatGPT in developing nations necessitates a substantial investment of time. In financially constrained countries, access to resources and opportunities for obtaining high quality education is limited ( Kshetri et al., 2023). Understanding the perspectives of teachers and students in developing nations on ChatGPT is essential to ensure its implementation aligns with their specific needs and circumstances. These findings inform the creation of effective integration strategies for settings with few resources, and ChatGPT has the potential to address educational gaps and offer customized learning opportunities. Nevertheless, the perspective of teachers and students at public and private universities in Bangladesh towards applying ChatGPT as an artificial intelligence tool for educational purposes remains largely unexplored.

To the best of the authors’ knowledge, more research is needed on ChatGPT in Bangladesh. Islam and Islam (2023) conducted a study to ascertain the advantages and disadvantages of implementing ChatGPT in educational settings in Bangladesh. To accomplish that goal, data were obtained from online sources and processed utilizing the noticing-collecting-thinking model. The outcomes of this AI strategy are divided into four categories: research, education, the development of individual skills, and social. Rahman and Watanobe (2023) conducted a study examining the potential benefits and risks that ChatGPT could have on education in Bangladesh. Furthermore, researchers investigated how ChatGPT improves students’ programming skills. They employed ChatGPT to conduct various coding-related experiments, such as generating algorithm pseudocode, rectifying code errors, and generating code based on problem descriptions. The generalizability of the study’s findings to teachers and students engaged in technology-related topics across different disciplines is limited.

Rahman et al. (2023b) carried out a study demonstrating the use of ChatGPT in academic research. They presented a practical example and offered suggestions. Published articles, websites, blogs, and visual and numerical artifacts were all used to gather the data for this study. The findings indicate that ChatGPT can serve as a valuable instrument for creating initial notions in scholarly studies. The literature synthesis, citations, problem descriptions, research gaps, and data analysis may present challenges for the researchers. These studies do not, however, focus on how teachers and students perceive the use of ChatGPT. Therefore, this study aims to ascertain how teachers and students at both public and private universities in Bangladesh perceive applying ChatGPT.

## Theoretical framework

**Figure 1.**
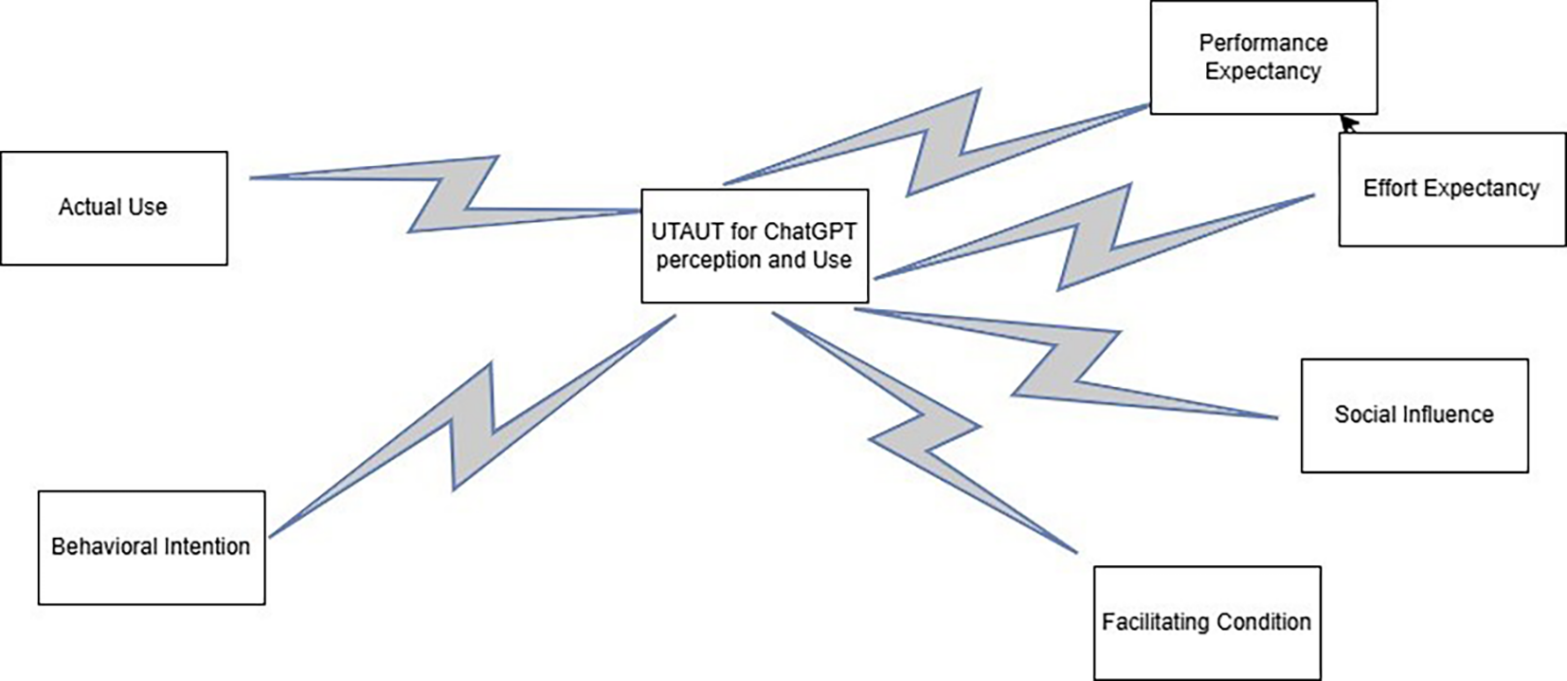
Theoretical framework of this study. This figure delineates the theoretical framework that guides this study. This framework is based on the Unified Theory of Acceptance and Use of Technology (UTAUT) model proposed by Venkatesh et al. (2003).

This study is grounded in the Unified Theory of Acceptance and Use of Technology (UTAUT) model, which posits four core constructs such as performance expectancy, effort expectancy, social influence, and facilitating conditions that are theorized to shape the acceptance and usage of technology ( Venkatesh et al., 2003). Performance expectancy is defined as the extent to which ChatGPT is perceived to improve educational outcomes, such as improving teaching efficiency for teachers or helping students during the learning process. Effort expectancy answers how easily teachers and students can leverage ChatGPT effectively during their studies. The social influence reflects the effect of peers, faculty, and institutional norms on the decisions of individuals to adopt ChatGPT. Finally, facilitating conditions are the resources and support at the educational institution that enable the use of ChatGPT, such as training or technical support. Along with these constructs, the UTAUT model added two vital outcomes: behavioral intention and actual use ( Rofiah & Suhermin, 2022). Teachers’ and students’ perceptions about ChatGPT and how they use ChatGPT were guided by behavioral intention and actual use outcomes to fulfill the objectives of this study ( Figure 1).

## Methods

This study followed a mixed method approach combining both quantitative and qualitative data to get a comprehensive understanding of teachers’ and students’ perception and usage of ChatGPT in social science faculty of both public and private unviersities in Bangladesh. This approach allow the researchers to generalize the perception and usage of ChatGPT by teachers and students along with the exploration of underlying views, experiences, and contextual factors of adapting ChatGPT. Heyvaert et al. (2013) argue that mixed-methods research is crucial because it combines quantitative data with qualitative data, which may provide a holistic understanding of complex phenomena. This strategy demands for a more in-depth exploration of findings. As a result, this study followed a mixed-method approach to generalize social science faculty teachers’ and students’ perceptions and use of ChatGPT in Bangladesh and also to comprehensively get an in-depth scenario of their experiences, strategies, and interactions. Two survey questionnaires were generated for both teachers and students. Each survey question contains twenty-six items and is prepared on a five-point Likert scale. In order to gain a comprehensive understanding of the perspectives of teachers and students from public and private universities in Bangladesh, interviews were carried out. As a theoretical framework, the UTAUT model helps to guide the preparation of questionnaires and the interpretation of the findings. However, no copyrighted survey items or scales from the proprietary UTAUT instrument were used in this study; rather, the model was adapted conceptually to develop data collection tools and also cited properly.

The survey items were developed based on the UTAUT constructs, including performance expectancy, effort expectancy, social influence, facilitating conditions, behavioral intention, and actual use. Items were generated through a review of prior UTAUT-based studies on technology adoption and refined to capture context-specific aspects of ChatGPT use in Bangladeshi higher education. The content validity of the two survey questionnaires was ensured through getting reviewed by three EdTech experts. As the questionnaires were in Bangla (the mother tongue of the participants), two language experts also validated the questionnaires. Based on their feedback, questionnaires were revised before piloting. Moreover, Cronbach’s alpha was utilized to assess the reliability of the instruments. Forty teachers and fifty-three students participated in the piloting of two surveys. While the students’ survey had a Cronbach’s alpha of 0.833, the teachers’ survey had a Cronbach’s alpha of 0.877. These results show that both questionnaires are highly reliable for gathering data.

Based on piloting feedback, a few items were reworded for clarity, redundant items were removed, and the overall structure of the two survey questionnaires was slightly modified to enhance the clarity of the tools. Pilot data were excluded from the final analysis to avoid bias in the main study. E-survey link was shared through institutional email lists, social media groups, and direct communication channels with teachers and students. A stratified sampling technique was used to collect survey data using Google Forms due to its wide accessibility, user-friendliness, and efficiency in organizing responses. The survey link was shared through institutional email lists, social media platforms, and direct communication with teachers and students. In terms of sample size, data were collected from the social science faculties of sixteen universities, comprising eight public and eight private universities. In total, survey data were collected from 402 teachers and 440 students across these social science faculties. However, after data cleaning, the dataset comprised 381 teacher records and 384 student records. Gender and other demographic factors were considered when collecting data, including data from 174 female teachers and 171 female students.

Then data was analyzed through IBM SPSSversion 27 ( IBM Corp, 2021) ( https://www.ibm.com/support/pages/downloading-ibm-spss-statistics-27). For researchers who seek an open-source alternative, can use, JASP for similar data analysis. JASP is an open-access software (available at https://jasp-stats.org/).

In terms of qualitative data, a convenient sampling technique was used for conducting in-depth interviews due to the accessibility and availability of teachers and students within the study timeframe. This convenience sampling allowed the inclusion of teachers and students from both public and private universities who were available and willing to provide detailed insights about their perception and use of ChatGPT. A total of 32 interviews were conducted, comprising 16 teachers and 16 students, with two teachers and two students from each university, ensuring equal gender representation at each university. The interviews were recorded with permission.

Research objectives were explained to the participants and commitment of conserving privacy of the participants in every aspects of the research were declared. Interviews were taken only those who willingly showed interest to participate in the study. Each interview lasted for 35 to 40 minutes. After collecting data, every recorded interview was transcribed verbatim. Next, all Bengali transcriptions were converted into English and was sent these to a Bengali and English language specialist to ensure the accuracy of the translation.

Researchers completed transcriptions after getting the translator’s input. The research employed the thematic qualitative data analysis technique to identify patterns and theme to understand the real situation deeply ( Braun & Clarke, 2006). By the six stages of data analysis outlined by Braun and Clarke (2006), all researchers first became familiar with the data by reading it separately, and then each researcher created preliminary codes from it. At this point, they adopted the strategy of “reading, reading, and reading again” ( Mertler, 2009, p. 141) to examine all narrative data. Researchers grouped related codes into new themes in the third step and then reviewed them again in the fourth. At the fifth step, several themes were identified, and mutually agreed-upon final themes were assigned names. A report was then generated. Since researchers transitioned from a factual understanding to a theoretical one, they employed an inductive approach to qualitative data analysis ( Graneheim, Lindgren, & Lundman, 2017). Direct quotes from teachers and students were used to highlight significant issues in the Findings section, thereby maintaining transparency and credibility. This approach allows participants’ voices to be authentically represented while facilitating the thematic interpretations.

**Table:** Examples of coding.

Codes	Sub-themes	Main Themes
Hear about ChatGPT, learn how to operate ChatGPT, do not know effective use of ChatGPT, Ability to operate ChatGPT all function, ChatGPT is wonderful, ChatGPT provide all answer, ChatGPT provide effective guideline, ChatGPT reduce creativity and critical thinking, do not like to use ChatGPT, ChatGPT provide wrong answer, Ethical dilemma	Knowledge of ChatGPT, Awareness about ChatGPT, Positive attitudes regarding ChatGPT, negative attitude regarding ChatGPT, Skills to operate ChatGPT	Knowledge, attitude and skill of using ChatGPT

## Findings

The following ( Table 1) demonstrates the demographic information of the teachers.

**Table 1.** Teachers demographic information.

		Count	Table N %
Types of University	Public University	202	53.0%
	Private University	179	47.0%
Gender	Male	207	54.3%
	Female	174	45.7%
	Others	0	0.0%
	No interest to share	0	0.0%
Position	Professor	39	10.2%
	Associate professor	62	16.3%
	Assistant professor	148	38.8%
	Lecturer	132	34.6%
Department	Economics	99	26.0%
	Political Science	80	21.0%
	Sociology	22	5.8%
	International Relationship	31	8.1%
	Mass Communication & Journalism	56	14.7%
	Public Administration	56	14.7%
	Anthropology	8	2.1%
	Population Science	16	4.2%
	Folklore	9	2.4%
	Social Work	4	1.0%
ChatGPT	Free version	302	79.3%
	Paid version	79	20.7%

Regarding the demography of teachers, it was found that public universities represented the majority (53%) among 381 participants, while 47% were from private universities. In terms of gender, 54% were male and 46% were female. Academically, assistant professors led by 38.8%, followed by lecturers of 34.6%, while associate professors and full professors made up 16.3% and 10.2%, respectively. In terms of academic discipline, Economics had the highest representation at 26%, followed by Political Science at 21%. Moreover, Mass Communication and Journalism, and Public Administration each accounted for 14.7% of the sample, while Anthropology had the lowest representation at only 1%. The majorities, 79.3% of teachers, have a free version of ChatGPT, and 20.7% have a premium version.

The following table ( Table 2) demonstrates the demographic information of the students.

**Table 2.** Student demographic information.

		Count	Table N %
Types of University	Public University	213	55.5%
	Private University	171	44.5%
Gender	Male	213	55.5%
	Female	171	44.5%
	Others	0	0.0%
	No interest to share	0	0.0%
Education level	Graduate	206	53.6%
	Post Graduate	178	46.4%
Education year	1st year	33	8.6%
	2nd year	82	21.4%
	3rd year	98	25.5%
	4th year	69	18.0%
	Masters 1st Semester	45	11.7%
	Masters 2nd Semester	36	9.4%
	Master’s Thesis Student	21	5.5%
Subject of Study	Economics	21	5.5%
	Political Science	34	8.9%
	Sociology	28	7.3%
	International Relationship	41	10.7%
	Mass Communication & Journalism	49	12.8%
	Public Administration	40	10.4%
	Anthropology	25	6.5%
	Population Science	25	6.5%
	Peace and Conflict	26	6.8%
	Social Work	27	7.0%
	Criminology	14	3.6%
	Women and Gender Studies	14	3.6%
	Development Studies	6	1.6%
	Television, Film and Photography	11	2.9%
	Education	22	5.7%
	Others	1	0.3%
Device	Laptop	192	50.0%
	Computer	58	15.1%
	Smartphone	131	34.1%
	Tab	3	0.8%
	Others	0	0.0%
ChatGPT	Free version	372	97.1%
	Paid version	11	2.9%

Survey data on student demographic showed that 55.5% of the student participants were from public universities and 44.5% were from private universities. The study includes the same gender distribution, with males constituting 55.5% and females 44.5%. Regarding the academic level, 53.6% of the respondents were graduate students, and 46.4% were post-graduate students. Mass Communication and Journalism was the most represented department in this study with 12.8% of students, followed by International Relationships (10.7%) and Public Administration (10.4%). Development Studies was the least represented department with1.6%. As for the usage of ChatGPT, 97.1% students utilized the free version, and merely 2.9% were premium users.

Teachers’ responses to the survey are presented in Table 3.

**Table 3.** Teacher response to the survey items.

Items	Mean	Std. Deviation
**Performance Expectancy (PE)**		
ChatGPT improves my capacity to teach complex issues efficiently	3.48	.803
Using ChatGPT enhances my efficiency in developing lesson plans and lectures	3.37	.796
ChatGPT assists me in developing more interesting teaching materials	3.51	.807
ChatGPT facilitates my research process, which encompasses concept formulation, literature review, methodology, and report writing	2.91	.858
ChatGPT enhances the quality of my study by providing rapid access to relevant materials	3.30	.937
**Effort Expectancy (EE)**		
I find that ChatGPT is user-friendly when developing instructional materials	3.58	.809
Incorporating ChatGPT with my teaching approaches requires minimal effort	3.48	.841
Understanding the use of ChatGPT for research activities is simple	2.83	1.024
I am confident of my ability to use ChatGPT for teaching and research	2.80	.955
**Social Influence (SI)**		
My colleagues support the use of ChatGPT in my teaching and research effort	2.69	.929
My institution supports the integration of ChatGPT within the educational environment	2.67	.904
The use of ChatGPT is becoming increasingly popular among teachers at my university	3.46	.944
**Facilitating Condition (FC)**		
I have the required technological resources to use ChatGPT in my teaching and research activities	3.32	1.142
My university offers adequate support for using ChatGPT as a teaching and research facility	2.49	.996
Institutional policies and guidelines for the ethical use of ChatGPT in educational contexts are established	2.47	1.017
I have financial support for using the Premium edition of ChatGPT	2.42	1.075
**Behavioral Intention (BI)**		
I plan to continue using ChatGPT for my teaching and research activities	3.62	1.023
I plan to increase my use of ChatGPT in the future for both teaching and research aims	3.68	.967
**Actual Use (AU)**		
I frequently utilize ChatGPT to develop teaching materials and lesson plans	3.73	1.089
I frequently utilize ChatGPT to assist with my research activities	3.74	.934
I frequently utilize ChatGPT to assign task to the students	2.87	1.080
I frequently consider the risk of plagiarism and ethical issues when using ChatGPT in my teaching and research	3.59	.944
I frequently verify the accuracy of the information supplied by ChatGPT	3.88	.931
I actively seek training opportunities to improve my use of ChatGPT for teaching and research	3.81	1.043
I am cautious about relying too heavily on ChatGPT for creativity in my teaching and research	3.82	1.015
I am cautious about potential challenges of using ChatGPT	3.70	.959

Teachers considered ChatGPT positively regarding its effective integration into enhancing teaching materials (M=3.51, SD=0.807), easy use (M=3.58, SD=0.809), and less effort required for incorporation into the teaching methods (M=3.48, SD=0.841). ChatGPT was frequently used to generate teaching materials (M=3.73, SD=1.089) and to check the accuracy of information (M=3.88, SD=0.931) but rated lower for helping with complex research (e.g., literature reviews and methods) (M=2.91, SD=0.858). The confidence level for using ChatGPT for research was relatively low (M=2.80, SD=0.955). These findings indicate that ChatGPT lacks reliability when applied in research purposes. Teachers also reported low levels of institutional support (M=2.49, SD=0.996) and policy guidance for ethical use (M=2.47, SD=1.017). Similarly, colleagues’ support (M=2.69, SD=0.929) and financial support for premium usage (M=2.42, SD=1.075) were presented as insufficient. These findings suggest that contextual and institutional support are essential for effective utilization of ChatGPT. Teachers intended to continue using ChatGPT (M=3.62, SD=1.023) and use it more (M=3.68, SD=0.967). They often thought about ethical risks (M=3.59, SD=0.944) and accuracy verification (M=3.88, SD=0.931), indicating a cautious optimism. They proactively sought training opportunities (M=3.81, SD=1.043) and were careful of excessive reliance on ChatGPT for creativity (M=3.82, SD=1.015). These findings indicate teachers’ concern about ethical risks and the overuse of ChatGPT.

Students’ response to the items is shown in Table 4.

**Table 4.** Student response to items.

Items	Mean	Std. Deviation
**Performance Expectancy (PE)**		
ChatGPT enhances my capacity to complete assignments more efficiently	4.31	.591
Applying ChatGPT enhances my understanding of complicated subjects and concepts	4.47	.625
ChatGPT improves the quality of my research papers and essays	4.04	.601
ChatGPT assists me in generating concepts for my research projects and assignments	4.44	.769
Employing ChatGPT enables me to accomplish work with greater rapidity and accuracy	4.47	.729
**Effort Expectancy (EE)**		
ChatGPT helps to finish my projects easily	4.05	.813
I can quickly acquire proficiency in utilizing ChatGPT for my research activities	4.21	.755
Using ChatGPT for academic study and research requires minimal effort	4.15	1.051
I am competent in utilizing ChatGPT for diverse learning activities	4.17	.745
**Social Influence (SI)**		
My peers suggest me to use ChatGPT for learning and research purposes	4.18	1.066
My teachers encourage the use of ChatGPT for assignments and research activities	3.17	1.412
The use of ChatGPT for learning purposes and assignments is increasingly popular among students in my class	4.07	1.114
**Facilitating Condition (FC)**		
I have the requisite technologies (smartphone, laptop, internet) to utilize ChatGPT for my studies	3.19	1.571
My university offers assistance and resources for utilizing ChatGPT in my studies	2.53	1.006
My university has established explicit standards for the ethical usage of ChatGPT in assignments and research	2.14	1.039
I have financial support for using the Premium edition of ChatGPT	2.39	1.261
**Behavioral Intention (BI)**		
I plan to keep using ChatGPT for my academic pursuits and research activities	3.49	1.035
I plan to increase my use of ChatGPT for future assignments and projects	3.41	1.116
**Actual Use (AU)**		
I regularly use ChatGPT to finish my projects and assignments	3.28	1.171
I frequently utilize ChatGPT to study for my courses and get ready for tests	3.75	.808
I frequently consider the possibility of plagiarism and moral dilemmas when utilizing ChatGPT for research and assignments	4.05	1.170
I frequently verify the information supplied by ChatGPT to make sure it is accurate for the assignments or research	3.68	1.104
I regularly use ChatGPT to prepare Power Point Presentation for academic purposes	4.07	1.078
I actively search for advice or tutorials to help me use ChatGPT more effectively for my academic work	3.89	.890
I try not to rely too much on ChatGPT to help me be creative with the assignments and research tasks	3.16	.959
I use ChatGPT to have language support while writing assignments	3.84	1.090

ChatGPT led to increased efficiency for students and better comprehension of the course material as students reported. It was beneficial with more complex subjects (M=4.47, SD=0.625) and ideas for project generation (M=4.44, SD=0.769). Furthermore, ChatGPT quickly and accurately helped with tasks (M=4.47, SD=0.729). Students often used it to complete assignments (M=4.31, SD=0.591) and prepare presentations (M=4.07, SD=1.078). Moreover, students reported ease of use when integrating ChatGPT into their academic activities, highlighting the low effort to use ChatGPT effectively (M=4.15, SD=1.051). They deemed themselves reasonably competent in using ChatGPT (M=4.17, SD=0.745) and frequently sought advice and tutorials from ChatGPT (M=3.89, SD=0.890). These findings indicate that students see ChatGPT positively because of support it provides for their academic activities. Moreover, Peer support for the use of ChatGPT was widespread (M=4.18, SD=1.066), but institutional support was perceived as insufficient. Clear ethical use standards (M=2.14, SD=1.039) and IT assistance provided by the universities (M=2.53, SD=1.006) received noticeably low ratings. Although indicating a sustained uptake of ChatGPT in academic settings, students expressed moderate to high intentions for continuous use (M = 3.49, SD = 1.035) and more future utilization (M = 3.41, SD = 1.116), they were cautious about overreliance (M=3.16, SD=0.959) and still focused on creativity and academic integrity. These findings suggest that although the students lack awareness about the ethical use of ChatGPT, they remain cautious for being overly dependent on it.

Table 5 shows the descriptive statistics according to six constructs.

**Table 5.** Descriptive statistics according to six constructs.

		N	Mean	Std. Deviation	95% Confidence Interval for Mean	
					Lower bound	Upper bound
PE	Public University Student	213	4.3587	.51155	4.2896	4.4278
	Private University Student	171	4.3287	.57461	4.2419	4.4154
	Public University Teacher	202	3.3485	.51497	3.2771	3.4200
	Private University Teacher	179	3.2715	.56392	3.1883	3.3547
	Total	765	3.8308	.74692	3.7778	3.8839
EE	Public University Student	213	4.1408	.62815	4.0560	4.2257
	Private University Student	171	4.1550	.67799	4.0526	4.2573
	Public University Teacher	202	3.1485	.62859	3.0613	3.2357
	Private University Teacher	179	3.1983	.63305	3.1050	3.2917
	Total	765	3.6614	.80467	3.6043	3.7185
SI	Public University Student	213	3.7762	.64873	3.6886	3.8638
	Private University Student	171	3.8499	.63674	3.7538	3.9460
	Public University Teacher	202	2.9373	.68096	2.8428	3.0318
	Private University Teacher	179	2.9441	.66337	2.8463	3.0420
	Total	765	3.3765	.78805	3.3205	3.4324
FC	Public University Student	213	2.5681	.78462	2.4621	2.6741
	Private University Student	171	2.5512	.82956	2.4259	2.6764
	Public University Teacher	202	2.6609	.70542	2.5630	2.7588
	Private University Teacher	179	2.6969	.71056	2.5921	2.8017
	Total	765	2.6190	.75904	2.5651	2.6728
BI	Public University Student	213	3.4366	.76137	3.3338	3.5395
	Private University Student	171	3.4737	.71180	3.3662	3.5811
	Public University Teacher	202	3.6807	.79380	3.5706	3.7908
	Private University Teacher	179	3.6089	.83675	3.4855	3.7324
	Total	765	3.5497	.78276	3.4941	3.6052
AU	Public University Student	213	3.7048	.61412	3.6219	3.7878
	Private University Student	171	3.7295	.58735	3.6409	3.8182
	Public University Teacher	202	3.6535	.59873	3.5704	3.7365
	Private University Teacher	179	3.6285	.63149	3.5353	3.7216
	Total	765	3.6789	.60841	3.6357	3.7221

The study uncovered distinct patterns among the six variables (PE, EE, SI, FC, BI, AU) highlighting differences in perceptions between the students and the teachers of different public and private universities. In terms of performance expectancy (PE), students at public universities had the highest mean (M=4.36, SD=0.58) for the usefulness of the system for improving performance. Students enrolled in private universities showed a mean score of 4.20 (SD=0.62). Public and private university teachers scored significantly less than the overall score (public university teachers: M=3.72, SD=0.64; private university teachers: M=3.60, SD=0.68). These findings suggest that ChatGPT contribute more to increasing students’ performance, whereas teachers from both public and private university perceive the contribution of ChatGPT in their own performance as limited. Furthermore, the effort expectancy (EE) trends mirrored those for Performance Expectancy (PE). Public university students obtained the highest (M=4.28, SD=0.57), followed by private university students (M=4.15, SD=0.59). The average score of the public universities teachers EE was 3.68 (SD=0.65), while the lowest average score, 3.55 (SD=0.70), belongs to the private universities teachers. These findings suggest that students can use ChatGPT with less effort compared to the teachers in both public and private universities. On the other hand, students from a private university showed the maximum mean score (M=3.85, SD=0.64) with the most significant perception of societal pressure to use the system, while public university students had a mean score of 3.72 (SD=0.63). Teachers scored lower on this construct— public university teachers scoring an average of 3.25 (SD=0.60) and private university teachers scoring an average of 3.18 (SD=0.65). In terms of social pressure, teachers reported less compelled to use ChatGPT than students in both public and private universities.

For the facilitating conditions (FC), scores were relatively low across all groups, reflecting a consensus on a lack of support/resources. The students from public universities scored slightly higher (M=3.12, SD=0.75) than those from private university students (M=3.05, SD=0.72). Teachers showed the lowest ratings: teachers in public universities (M=2.95, SD=0.68) and private universities (M=2.90, SD=0.70). However, unlike other constructs, teachers had a higher behavioral intention (BI) than students. The highest mean score (M=4.10, SD=0.65) was identified for public university teachers, followed by private university teachers (M=4.00, SD=0.68). Still, public university students scored an average of 3.88 (SD=0.66), and private university students scored an average of 3.80 (SD=0.68). Lastly, regarding actual use (AU), having little variability, all groups were impressively consistent in how they scored. Public university teachers averaged 3.75 (SD=0.58), slightly ahead of private university teachers (M=3.70, SD=0.60). Students showed similar relative scores, with average scores of public university students of 3.68 (SD=0.63) and private university students of 3.65 (SD=0.64). In both public and private universities, teachers and students need institutional support to use ChatGPT and they have high behavioral intention to use it in future.

**Table 6.** One way ANOVA statistics.

ANOVA						
		Sum of Squares	df	Mean Square	F	Sig.
PE	Between Groups	204.717	3	68.239	234.430	.000
	Within Groups	221.515	761	.291		
	Total	426.232	764			
EE	Between Groups	182.140	3	60.713	147.827	.000
	Within Groups	312.547	761	.411		
	Total	494.687	764			
SI	Between Groups	144.782	3	48.261	111.399	.000
	Within Groups	329.683	761	.433		
	Total	474.465	764			
FC	Between Groups	2.781	3	.927	1.613	.185
	Within Groups	437.395	761	.575		
	Total	440.175	764			
BI	Between Groups	7.806	3	2.602	4.302	.005
	Within Groups	460.306	761	.605		
	Total	468.112	764			
AU	Between Groups	1.167	3	.389	1.051	.369
	Within Groups	281.640	761	.370		
	Total	282.807	764			

The one-way ANOVA indicates that the differences between groups are significant for the constructs of Performance Expectancy (PE), Effort Expectancy (EE), and Social Influence (SI) (p < .001 for each. PE between-group data showed substantial variation (Sum of Squares = 204.717, F(3, 761) = 234.430, p < .001), indicating substantial group-level differences. EE also showed significant differences (Sum of Squares = 182.140, F(3, 761) = 147.827, p < .001) and SI (Sum of Squares = 144.782, F (3, 761) = 111.399, p < .001). Behavioral Intention (BI) was significantly changed, but less strong effect (Sum of Squares = 7.806, F(3, 761) = 4.302, p = .005). However, no statistically significant differences were found for Facilitating Conditions (FC) (F(3, 761) = 1.613, p = .185) or Actual Use (AU) (F(3, 761) = 1.051, p = .369), indicating that attitudes for these categories were consistent across groups. The results deliver a comprehensive overview of multifaceted heterogeneity in users’ attitudes and intentions across multiple cohorts highlighting common challenges in favorable conditions and actual usage behavior, particularly in terms of perceived usefulness, ease of use, and social influence.

Tukey’s HSD post hoc analysis showed significant group-level differences across constructs. Performance Expectancy (PE) and Effort Expectancy (EE): Students in both public and private universities scored higher than teachers (p < .001), where the highest scorers were the students of public universities. These findings suggest that students perceive ChatGPT as valuable tool for increasing their academic performance and they acclaim its easy interface to use while teachers are more concern about ethical use of ChatGPT and inaccurate information provided by ChatGPT which impact on their usage of ChatGPT. This gap highlights the need for students to focus on the ethical use of ChatGPT, while teachers need training to use it effectively. Students outperformed teachers (p < .001) based on Social Influence (SI), whereas private university students performed the best. These findings suggest that students’ use of ChatGPT is influenced by their peer networks; however, without institutional guidance, it may lead to excessive and improper use of ChatGPT. In contrast, teachers may face weaker peer pressure to use ChatGPT. By Behavioral Intention (BI): the teachers outscored students, and public university teachers outscored private university teachers. However, the difference was not statistically significant. These findings underscore teachers’ willingness to utilize ChatGPT for teaching and research purposes in the future, provided their concerns regarding ethical use and potential inaccuracies are addressed. These findings indicate that social factors influence students’ use of ChatGPT, whereas teachers’ use of ChatGPT is driven by their behavioral intention. Peer-driven initiatives are necessary for teachers’ collective engagement, while a clear institutional policy is needed for students to use ChatGPT responsibly.

## Findings from Qualitative analysis

### Demographic profile of the participants

**Table 7.** Teachers profile.

ID	Name (Pseudonym)	Gender	Teaching experience (Years)	University type
T1	Rahul	Male	5	Public
T2	Shilpi	Female	12	Public
T3	Baizid	Male	14	Private
T4	Sharmin	Female	3	Private
T5	Sanjoy	Male	23	Public
T6	Fahmida	Female	10	Public
T7	Rahim	Male	7	Private
T8	Sumaiya	Female	9	Private
T9	Kamal	Male	13	Public
T10	Aleya	Female	20	Public
T11	Kuddus	Male	2	Private
T12	Sokina	Female	11	Private
T13	Jabber	Male	15	Public
T14	Jorina	Female	3	Public
T15	Sumon	Male	5	Private
T16	Kulsum	Female	9	Private

**Table 8.** Students profile.

ID	Name (Pseudonym)	Gender	Level of study	University type
S1	Morjina	Female	Masters	Private
S2	Babu	Male	Honors (2 ^nd^ year)	Private
S3	Hymonti	Female	Honors (4 ^th^ year)	Public
S4	Rabbi	Male	Honors (1 ^st^ year)	Public
S5	Nahar	Female	Masters	Private
S6	Milon	Male	Honors (3 ^rd^ year)	Public
S7	Baby	Female	Honors (2 ^nd^ year)	Private
S8	Arafat	Male	Masters	Private
S9	Hena	Female	Honors (1 ^st^ year)	Public
S10	Mukul	Male	Masters	Public
S11	Asma	Female	Honors (4 ^th^ year)	Private
S12	Motin	Male	Masters	Private
S13	Tanni	Female	Honors (2 ^nd^ year)	Public
S14	Shahid	Male	Honors (1 ^st^ year)	Public
S15	Naju	Female	Masters	Private
S16	Mollik	Male	Honors (3 ^rd^ year)	Private

**Figure 2.**
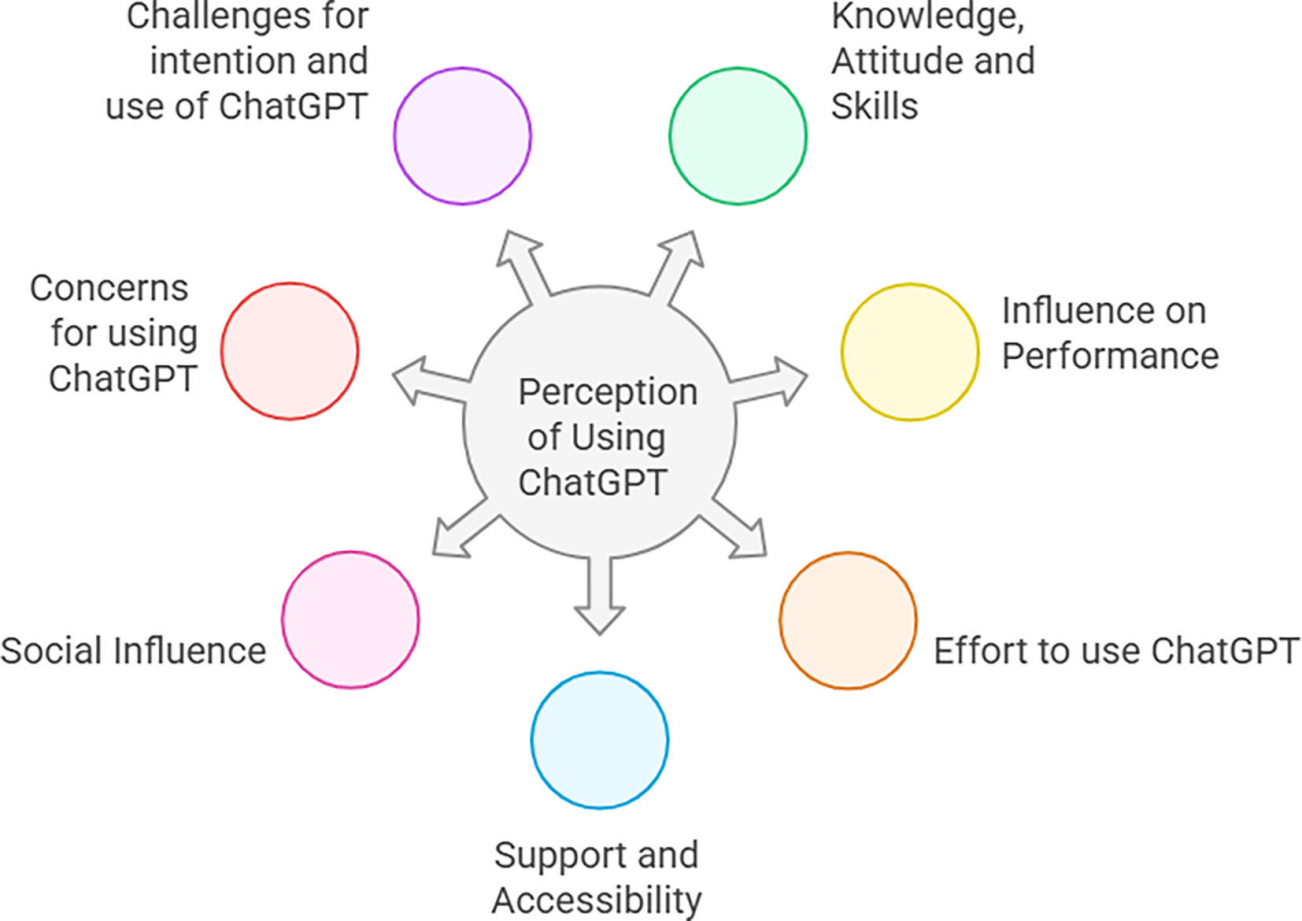
Comprehensive framework from thematic analysis. This figure illustrates a comprehensive framework of this study, which emerged from the thematic analysis of qualitative data from 16 teachers and 16 students in in-depth interviews. It highlights seven key themes, including knowledge, attitude, and skills; influence on performance; effort to use ChatGPT; support and Accessibility; Social Influence; concerns for using ChatGPT; and challenges for intention and use of ChatGPT.

The following figure ( Figure 2) demonstrates a comprehensive framework developed from thematic analysis of qualitative data.

### Knowledge, attitude and skill of using ChatGPT

For students and teachers in Bangladesh, using ChatGPT is an unconventional idea. Both public and private university teachers claim that they need to become more acquainted with ChatGPT as their proficiency in using ChatGPT for teaching is average. Many university students, both public and private, have the necessary knowledge and abilities to make successful use of ChatGPT. The majority of them claim to be sufficiently knowledgeable about ChatGPT. These involve interacting with artificial intelligence as well as navigating and using ChatGPT. With their ability to command and instruct, they have the necessary skills and knowledge to extract accurate data from AI efficiently. Most of them used YouTube tutorial videos to learn how to use ChatGPT. One public university (S14) student said,

“I am proficient in utilizing ChatGPT without any difficulty. I employed ChatGPT without undergoing any training. I acquired proficiency in using new programs using YouTube. I utilize it as a means of assistance when I encounter difficulties.”

A private university student (S5) also mentioned,

"It is easy to use ChatGPT. Anyone with a basic idea about technology can operate ChatGPT easily. However, to get a genuine answer, it is crucial to know how to ask questions effectively to ChatGPT. So, these questioning skills are crucial."

However, ChatGPT is highly regarded by both students and teachers at private universities. Private university students and teachers have shown great interest in and excitement about using ChatGPT. Using ChatGPT, users can quickly receive answers to their questions and verify the accuracy of their information.

A private university teacher (T4) said,

“ChatGPT and other AI have a very positive impact on our education sector. I consider ChatGPT positively, but everyone needs to be careful to maintain academic integrity.”

Some teachers in public universities, on the other hand, have a negative outlook. Some teachers have reported negative consequences for teachers and students due to using ChatGPT. Some teachers believe using ChatGPT in the classroom can jeopardize current teaching strategies and possibly reduce teachers’ efficacy. Furthermore, several teachers contend that ChatGPT impedes students’ intellectual growth. A public university teacher (T10) said,

“ChatGPT is fostering a sense of laziness among our adolescent learners. Students are currently quickly accessing information through ChatGPT, diminishing their reliance on cognitive abilities. Consequently, students’ creativity and mental abilities are being impaired.”

### Influence on performance

ChatGPT is viewed favorably by faculty members at private universities when it comes to creating course materials. According to several teachers, ChatGPT is a tool that helps them better comprehend any content and improves their knowledge of the material. Teachers at public universities point out that ChatGPT’s ability to offer thorough information on a range of topics is limited. Though the teachers acknowledged that relying solely on ChatGPT for developing classroom presentations is inappropriate, they foud it convenient to having a comprehensive review of the topic. Both teachers in public and private universities acknowledge that ChatGPT enhances their capacity to address students’ inquiries, elucidate concepts, generate stimulating discussion topics, perform language translations, and generate personalized learning materials such as quizzes and flashcards. It guides the most effective teaching strategies or tactics for a specific subject. Moreover, it facilitates designing lessons and provides comprehensive expertise in several subjects, positively impacting their professional competence. A teacher from a private university (T16) remarked,

“Previously, I needed a comprehensive grasp of several topics. However, I now endeavor to enhance my knowledge by utilizing ChatGPT, which has resulted in improved academic achievement. I have learned the appropriate techniques to employ for different types of content.”

Through ChatGPT, faculty members at public and private universities can easily perform research activities such as idea generation, setting research objectives, developing theoretical frameworks, evaluating literature, and developing techniques. However, most teachers are hesitant to use ChatGPT in their domain because of the inaccurate and deceptive information it produces. The links or sources that ChatGPT offers are not trustworthy. Several academics argue that ChatGPT’s citations to DOI (Digital Object Identifier) are inaccurate. A public university teacher (T1) said,

“For basic understanding about a topic, ChatGPT is great. It can also guide which process we can follow in our research, but it provides a lot of wrong information which can cause a disastrous effect in research and academic outcomes.”

On the other hand, both students at public and private universities concur that it enhances their academic outcomes. With ChatGPT, users may easily comprehend the subject matter and obtain suggestions for assignments or presentations, directly influencing their academic achievements. It enhances students’ learning efficiency, hence facilitating the attainment of their learning goals. In addition, numerous students at public universities assert that they can utilize it to disseminate content through different approaches. Currently, students in the classroom exhibit heightened receptiveness and increased engagement compared to previous instances. A student of a public university (S9) claimed,

“Utilizing ChatGPT has dramatically enhanced our learning efficacy. Now, it is straightforward to acquire a comprehension of the predetermined syllabus. Consequently, our active engagement in the classroom learning process has intensified.”

Besides, both private and public university students concur that it facilitates their study and research. They increasingly rely on ChatGPT to obtain answers to a wide range of issues. They acquire a diverse range of knowledge from it. It facilitates the development of their essays, reports, and compositions for monographs. They get advantages from it while creating presentation slides and collaborating in groups. They also derive benefits from it for their thesis. A student of a private university (S15) said,

“The little time given for assignments poses a significant challenge in creating a well-crafted task within a tight timeframe. With the assistance of ChatGPT, I can now efficiently generate well-informed and suitable assignments in a short period.”

Teachers and students at public and private universities point out the different kinds of assistance and guidance that are accessible when using ChatGPT. They spend the same time looking through numerous search engines to read stuff. It also helps them overcome the challenges presented by the English language, simplify complex ideas, and create educational materials. It assists people in improving their subject matter, pedagogical, and technological proficiency. Teachers and students at both public and private universities have shown their support for ChatGPT. ChatGPT is reportedly expanding its knowledge base and improving its research and analytical skills. Several students highlight that ChatGPT’s constant availability allows them to seek assistance beyond regular class hours. Students also noted that ChatGPT has the potential to assist them in addressing a problem by considering multiple perspectives, thus enhancing their capacity for critical and creative thinking from different angles. A student of a private university (S8) said,

“ChatGPT has expanded the scope of my learning. The processing time for every task is minimal. Each time I inquire, I promptly receive a highly rational explanation, enhancing my cognitive abilities.”

### Effort to use ChatGPT

All participants assert that the ChatGPT interface is user-friendly. Consequently, all individuals can use ChatGPT without encountering significant challenges. They can function and traverse effortlessly as a result of a straightforward interface. All participants acknowledge that ChatGPT provides prompt responses. It uses simple and easily understandable language. It endeavors to illustrate any idea using examples and in a straightforward manner. A student from the public university (S3) said,

“When I used ChatGPT for the first time , I thought it might be complex. As I am not handy to technology, but I found simple steps to use it. I just need to provide specific command to get sufficient answer.”

However, teachers of both public and private universities considered the interface of ChatGPT user friendly . Though it is easy to operate, they are concerned for using it in academic research purpose due to lack of specific guideline to use it in the research. One of the private university teachers (T11) mentioned,

“It is good to use ChatGPT to get a basic idea about research. It even helps guide the theoretical framework. However, my concern is how I will use it in the study, as I do not know whether specific guideline prevails or not to use it. As a result, I have not tried to use it in research. I use it to get some basic ideas.”

### Support and accessibility

Participants opine they use multiple electronic devices and operating systems (smartphone, tablet, laptop, desktop computer) to access ChatGPT. However, they require strong networks and speedy internet connections. Sometimes, network problems can stop them from using ChatGPT. Everyone agrees that ChatGPT also works in other languages, but its answers in English are always better. It fails to give correct answers in Bangla in some cases and misinterprets commands sent in Bangla at times. In addition, study participants asserted that they could not use the ChatGPT Plus subscription edition. All the participant did not get any institutional support to use ChatGPT Given the budgetary constraints, many teachers and students in Bangladesh need help to afford the expensive fees associated with premium usage—consequently, users of the complimentary version express dissatisfaction with the few resources provided by ChatGPT. A private university student (S12) said,

“I use the accessible version of ChatGPT. I cannot buy the premium version as it is costly. Sometimes, I do not get answers from ChatGPT about my queries.”

A teacher of public university (T14) said,

“I think to cope with present era, we cannot ignore AI like ChatGPT. We need to get access to be updated ourselves by using this tools but our financial barrier impedes us to use ChatGPT-4. Universities can support in this sector.”

### Social influence

Most of the students from public and private universities admitted that they were inspired to use ChatGPT by their peers. Peers who are good at using ICT, they mainly influence other students to use ChatGPT for assignment, presentation slide, and various queries. A private university student (S16) said,

“When I struggled to prepare my assignment, my classmate suggested using ChatGPT. For his inspiration, I signed up for the ChatGPT free version, and he was waiting for me. I got everything that I needed. I embraced my friend for his suggestion.”

Few students get inspiration from teachers to get basic ideas of different complex term, but teachers are not influenced by their colleagues. Most of the teachers of public and private universities admitted that they were not influenced by their colleague to use ChatGPT or other AI tools.

A public university teacher (T7) said,

“We are swamped taking classes as it is a teaching university. We do not have time to discuss innovations or things like that. If we meet, we chitchat to relax. Hardly academic discussion we do.”

However, a private university teacher (T13) mentioned,

“My colleagues and I sometimes discuss the use of ChatGPT. If anyone becomes confused about any issue, others suggest seeking help from ChatGPT.”

### Concern for using of ChatGPT

Most faculty members know its reliability, indicating that they possess an elementary awareness of its advantages and disadvantages. Teachers have noted that it sometimes provides inaccurate information. The system autonomously generates information; however, its information is unreliable. It generates random and imprecise points of reference. Occasionally, it needs to more comprehend the context of a query, leading to responses that appear irrelevant or unrelated. Furthermore, it generates stuff that is hypothetical or fictional. In addition, several educators express dissatisfaction with its practice of distributing identical content to multiple users, which diminishes their confidence in its effectiveness. In this regard, a teacher of a public university (T2) stated,

“I have reservations about ChatGPT due to its tendency to disseminate inaccurate content and its need for more transparency on the sources of information. I solely utilize it for grasping the fundamentals, as I need clarification regarding the reliability of the content.”

However, all students admit that they use ChatGPT without fully understanding its workings. They never doubt the precision of ChatGPT. As digital technologies become increasingly prevalent in classrooms and other learning environments, students often need to pay more attention to their limitations and potential for error. One public university student (S14) mentioned,

“I am not worried about the reliability of the AI information because it makes my life more enjoyable. I finished my assignments very quickly with the help of ChatGPT.”

Issues regarding the ethical implications of ChatGPT are expressed by all university teachers, whether from private or public universities. Using material that requires author acknowledgment might be difficult for teachers due to ethical constraints. Several academics state that there is no standard procedure for keeping track of data produced via ChatGPT. Therefore, more extended data retention periods give rise to privacy and data security issues. In addition, teachers at both public and private universities note that students require more excellent education regarding the moral implications of ChatGPT. While additional plagiarism detection methods are needed and their use is limited, teachers cannot evaluate the authenticity of their students’ assignments, reports, and monographs. As a result, using ChatGPT requires adherence to institutional rules and policies. Teachers at private and public universities acknowledge the potential disadvantages of ChatGPT in educational settings and take steps to deal with them. They express concerns over excessive dependence on ChatGPT. They are concerned that relying on it too often may hinder their capacity to learn autonomously and solve difficulties. Teachers at private universities complain that ChatGPT is fostering complacency among them. Teachers employ ChatGPT to confine their expertise inside a specific domain, unlike their predecessors, who would amass their knowledge by perusing several books, publications, and articles. Excessive reliance on ChatGPT may undermine teachers’ capacity for critical thinking and independent inquiry. In this regard, a teacher at a private institution (T8) stated that,

“ChatGPT has fostered our reliance on technology. Previously, we relied on diverse sources to acquire knowledge, while now we can promptly get information. Its detrimental impact outweighs its beneficial effects. We are diminishing our cognitive abilities.”

Teachers are reluctant to exclusively utilize technology for their advantage due to concerns for their students. Most university faculty members acknowledge the potential danger of excessive reliance on technology as students grow increasingly intertwined with artificial intelligence. Consequently, students’ capacity for critical thinking and problem-solving is reduced. The utilization of ChatGPT by students is hindering their potential to cultivate autonomous thinking and originality, as well as their aptitude to engage with people, communicate proficiently, and foster commendable reading and writing practices. One of public university teachers (T5) remarked,

“Previously, students acquired knowledge by engaging in peer discussions. Nevertheless, their current utilization of ChatGPT for information retrieval impedes their consciousness development.”

However, university students and teachers concur that educational establishments can mitigate the adverse impacts by fostering critical thinking, digital literacy, well-rounded integration, and ethical considerations. Furthermore, they believe continuous assessment and adjustment are vital to ensure that AI improves rather than diminishes these tools. It is necessary to develop and enforce specific policies for utilizing ChatGPT in educational and research endeavors to mitigate potential adverse consequences arising from its use over an extended period **.**

### Challenges for intention and use of ChatGPT

The majority of participants encounter diverse challenges when using ChatGPT. Several teachers emphasize the limitations of ChatGPT, such as its tendency to present biased or inaccurate material, its need for more subject-specific expertise, and its tendency to provide superficial or shallow information. Teachers need help to place confidence in the content produced by ChatGPT due to its failure to attribute credit to the original authors of the information which decrease their intention to use ChatGPT in research purpose. Evaluating the authenticity and novelty of assignments provided by students through ChatGPT also poses difficulties for teachers. Academic integrity is compromised when students exploit ChatGPT to generate plagiarised content for their assignments and examinations. Teachers face challenges in fostering critical and profound engagement among their students, as they can quickly obtain answers using ChatGPT. Considering the current prevalence of student attachment to ChatGPT, all teachers unanimously agree that ChatGPT poses some challenges. In this regard, a public university teacher (T9) stated,

“Within our educational system, a significant proportion of the students enroll in higher education institutions to acquire a degree, which serves as a means to ensure future work opportunities. They continuously assess their efficiency in earning degrees. These students are fortunate to have access to ChatGPT but they are reluctant to attend classes.”

While utilizing ChatGPT, students face many challenges in conjunction with lecturers. Students also met the language intricacy of ChatGPT, which hindered their comprehension of the information it conveyed. Most students held unfavorable views on the exorbitant cost of ChatGPT’s premium edition, as it was outside their financial means. ChatGPT users may encounter technical difficulties such as system crashes, malfunctions, network challenges, or internet connectivity problems.

## Discussion

Bangladesh, as a developing nation, is undergoing through a transformation since it has been trying to integrate technology into several sectors, including education. The swift progress in Natural Language Processing technology has allowed educational institutions to consider incorporating innovative ways of instruction and learning. Teachers and students in the tertiary educational institutions at Bangladoesh use ChatGPT for their academic needs. According to this study, students of both public and private universities use ChatGPT as a highly immersive platform, demonstrating a high-performance expectation across the platform. This finding aligns with the research done by Limna et al. (2023), which found that students think ChatGPT helps them learn complex subjects. However, performance expectancy scores were lower for teachers than students at both public and private universities. This discrepancy can result from teachers’ cautious views regarding ChatGPT’s effects on learning environments. According to study by Kasneci et al. (2023), teachers are more suspicious about the practical usage of ChatGPT in teaching and research, even when students adapt it to some degree due to possible benefits. According to the effort expectancy results for both student groups of this study, ChatGPT was user-friendly. This is in line with Ollivier et al. (2023), who highlight how easy it may be for students to use AI tools. Both public and private university teachers’ have moderate level effort expectancy scores that also reflected in Sok and Heng (2023) study while they resonates with the need for professional development that can boost confidence and ease of use of AI.

Social influence is an important factor for ChatGPT usage. The findings show a moderate social influence on students’ use of ChatGPT, indicating that peer support plays a significant role in students’ engagement with AI tools. These findings support earlier research suggesting that classroom social dynamics significantly influence students’ use of technology ( Winstone & Boud, 2022). On the other hand, both teacher groups scored lower on social influence, indicating that their institutions and peers don’t encourage them to use ChatGPT. Dawa et al. (2023) emphasize that positive feedback from colleagues increases teachers’ ChatGPT usage and encourages them to adopt it. ChatGPT usage also depends on some facilitating conditions. All teachers and student groups have identified the availability of technology resources and the institution’s support for using ChatGPT in an educational setting as barriers and poor facilitating conditions. This is consistent with studies by Limna et al. (2023) that highlight the need to have sufficient infrastructure and support mechanisms to ensure successful technology integration. These results also highlight a critical area for educational institutions to address since the potential benefits of AI technologies for teaching and learning might be seriously hampered by a lack of supportive environments.

Teachers exhibited a stronger behavioral intention to use ChatGPT than their students, although students in both groups expressed a modest intention to use ChatGPT in the future. This means teachers are more committed to employing these tools in their practices, even when students desire to use them. This result is consistent with studies that indicate teachers are beginning to recognize the benefits of using AI tools to improve their teaching ( Ollivier et al., 2023). However, it also emphasizes how crucial ongoing assistance and training are to guarantee this commitment. According to the actual use scores of this study, both groups of students frequently utilize ChatGPT for various academic assignments, which highlight how actively they engage with AI technologies in their studies. This is also consistent with research by Limna et al. (2023) that shows students want to use technology to improve their educational experiences. Though their ratings were lower than those of students, teacher usage rates were also quite high, suggesting impediments to integration into teaching roles that are in line with Sok and Heng’s (2023) findings.

In addition to the various benefits of employing ChatGPT for academic purposes, a few teachers agree that the utilization of ChatGPT carries specific adverse ramifications for both teachers and students. Excessive comfort in working environments may hinder pupils’ intellectual maturation by suppressing their need to engage in critical thinking. Moreover, Bai et al. (2023) found that an overabundance of ChatGPT usage leads to a diminished ability to engage in critical thinking. ChatGPT has been identified as the cause of decreased memory recall. Furthermore, it might also hinder the professional growth of educators. Teachers at private universities utilize ChatGPT to enhance their lecture preparation and expand their expertise in various subjects. Conversely, teachers at public universities believe that ChatGPT’s knowledge base is limited and primarily receptive rather than comprehensive. Garg et al. (2023) and Spennemann (2023) claim that though ChatGPT is receptive to information, its capacity to provide extensive knowledge on certain subjects is very constrained.

According to Castillo et al. (2023), most students utilize ChatGPT due to its rapidity and precision, which benefit their learning process. Being a user from Bangladesh, individuals occasionally need some help due to the limitations of ChatGPT in providing proper responses in the Bangla language. It frequently misinterprets orders given in Bangla, which aligns with the research conducted by Kolar and Kumar (2023) on Hindi, Telugu, and Kannada. Furthermore, the requirement of a ChatGPT Plus subscription presents an additional obstacle to utilizing ChatGPT. Bangladesh is classified as a lower-middle-income country. In Bangladesh, there are lots of resource constraints to use online for teaching-learning like network issues, internet connectivity and speed, financial barriers ( Rahman et al., 2023a). Hernandez (2019) posits that the financial constraints faced by hinder its ability to adapt technology in several sectors where it is required effectively. Similarly, most teachers and students in Bangladesh need help to afford the expensive fees associated with premium usage, primarily due to budgetary constraints. Consequently, consumers encounter numerous problems when using the free edition of ChatGPT.

ChatGPT has been found to exhibit inaccuracies or misinformation. Since it can autonomously provide information that could be more reliable, furthermore, it occasionally shows a deficiency in comprehending the context of a query, leading to the provision of irrelevant responses. Moreover, it generates content that is speculative or fictional. Nevertheless, despite all the constraints, the students use ChatGPT without considering its drawbacks. As students get more familiar with digital tools, they may need to pay more attention to the increasing prevalence of faults in academic procedures. However, teachers have limitations in assessing the authenticity of their students’ assignments, reports, and monographs because of the absence of plagiarism detection systems and their limited accessibility. Consequently, adhering to institutional regulations and guidelines is necessary to utilize ChatGPT. Limna et al. (2023) have highlighted the necessity of establishing an institutional policy to mitigate the disadvantages associated with using ChatGPT.

Teachers possess knowledge of the potential limitations of ChatGPT when used in educational settings. Despite their continual usage, they have concerns that it may hinder their capacity to learn autonomously and resolve issues. Contemporary educators utilize ChatGPT to confine their expertise to a particular domain, unlike their predecessors, who would amass their information by perusing several books, publications, and articles. The overreliance on ChatGPT may undermine teachers’ capacity for critical thinking and independent inquiry. Teachers express concerns about their students’ overreliance on technology and artificial intelligence. Consequently, students’ cognitive skills, such as critical thinking and problem-solving, may be impaired. Nevertheless, there is a consensus among both educators and learners that educational establishments can mitigate the adverse consequences by fostering critical thinking, digital literacy, well-balanced incorporation of AI technology, and ethical considerations. Explicit policies must be formulated and executed to utilize ChatGPT in educational and research endeavors.

## Conclusion

This study endeavors to investigate the perception and use of ChatGPT by social science faculty teachers and students at public and private universities in Bangladesh for instructional purposes. This study reveals several noteworthy concerns that require further contemplation and examination. The study findings suggest that students of both public and private universities have higher performance expectancy and effort expectancy compared to their teachers. Teachers hold divergent perspectives on using ChatGPT, with some displaying enthusiasm while others expressing skepticism. Nevertheless, students at both types of universities view ChatGPT favorably for a range of educational endeavors and learning prospects, such as improved knowledge and research capabilities. Students from both private and public universities affirm that utilizing ChatGPT improves their learning outcomes and positively impacts their academic achievement. ChatGPT is also helpful for teachers. It enhances their teaching efficacy by assisting in class planning and providing comprehensive expertise on the subject matter. In addition, students of both public and private universities are more socially influenced by their peer networks, while teachers face weaker peer pressure but have higher behavioral intention to use ChatGPT. Despite these differences, neither teachers nor students are satisfied with the facilitating conditions of using ChatGPT, such as institutional support. For example, due to the exorbitant cost of ChatGPT Premium, users often resort to using the free version, which offers limited functionalities. Robust networks and high-speed internet are essential for enhancing the accessibility of ChatGPT.

Furthermore, the efficiency of the English language and the limitations of other languages pose a significant obstacle to its effective utilization. This study also investigates the apprehension regarding the dependability of information offered by ChatGPT due to potential misinterpretation of queries. In addition, teachers express concerns over the ethical implications of utilizing ChatGPT and the potential for excessive reliance on it, students from both public and private universities still need to be made aware of these difficulties. Teachers concern about ethical use and over-reliance on ChatGPT reflect global debate on ChatGPT impact on academic integrity, assessment authenticity, and critical thinking ability (Kasenci et al., 2023; Rudolph et al., 2023). Besides, students limited awareness about ethical and responsible use of ChatGPT highlights urgent institutional policy to use ChatGPT. This study findings prioritize to eliminate barriers, including addressing ethical concerns, to ensure equitable access to ChatGPT and harness its potential to enhance the quality of teaching, learning, and research in higher education. The findings of this study will assist in other developing and underdeveloped countries with socio-economic and cultural contexts similar to Bangladesh’s, contributing to their higher education systems. In addition, this study will contribute in global literature on GenAI ethics in education. However, this study only focused on the social science faculty, teachers, and students of multiple universities in Bangladesh, so the findings may not reflect the holistic perspective of ChatGPT usage among all demographic user categories. Focusing on a single set of users limits the generalizability of the findings to a larger population because users from other areas of interest or geography may have completely different experiences and perceptions. Future studies could address these challenges and investigate ethical awareness, policy implication, and leadership role to proper use of ChatGPT.

## Ethical approval statement

This study adhered to the ethical guidelines in the Declaration of Helsinki and received approval from the Institute of Education and Research Human Research Ethics Committee (IERHREC) with approval number 2023/01 on 10/02/2023. The protocol for the study was reviewed to ensure its adherence to ethical norms governing research involving humans. This review encompassed the protocol’s aims, methodology, and informed consent processes. All individuals who took part in this study provided their consent before participating, and their confidentiality and privacy rights were protected during every stage of the research procedure. All potential risks to the participants were minimized.

## Informed consent

Researchers informed the study objectives and privacy conservation issue to the participants and provided written consent letter to the participants. After getting consent from the participants, researchers collected data.

## Authors contribution statement

Conceptualization and planning by 1 ^st^ author, tools development by 2 ^nd^ author, data collection and data analysis by 3 ^rd^ and 4 ^th^ author; writing introduction, literature review, and implication by 1 ^st^ author, theoretical framework and methodology by 2 ^nd^ author, discussion by 3 ^rd^ author, conclusion by 4 ^th^ author; supervision, review and editing by 1 ^st^ and 2 ^nd^ author. All authors read the manuscript thoroughly and agreed to submit and publish in this journal.

## Data Availability

Figshare: *Teachers’ and students’ use of ChatGPT at Social science faculty in the public and private Universities of Bangladesh*, https://doi.org/10.6084/m9.figshare.28351223 ( Rahman et al., 2025a) This study dataset entitled consists of the following three data: Dataset1—This data set consists of 402 teacher survey responses from social science faculty of public and private universities about their perceptions and practices of using ChatGPT. Dataset2—This data set consists of 440 student survey responses from social science faculty of public and private universities about their perceptions and practices of using ChatGPT. Dataset3—This dataset consists of qualitative data from 16 teachers and 16 students in-depth interviews. These data are available under the license CC BY 4.0, which allows unrestricted use and reproduction. To ensure confidentiality and privacy, all data sets have been attached after hiding participants’ identities. **Figshare:** *Supplementary file for ChatGPT use of social science faculty teachers and students.* 10.6084/m9.figshare.30025888.v1 ( Rahman et al., 2025b) This supplementary file consist of five data files as an extended data: Survey questionnaire: Two survey questionnaires and in-depth interview protocol used for teachers and students of the social science faculty of both public and private universities of Bangladesh in this study. Moreover, a consent form and an example of coding have also been provided. These data are available under the license CC BY 4.0, which allows unrestricted use and reproduction. To ensure confidentiality and privacy, all data from supplementary file have been attached after hiding participants’ identities.

## Software availability statement

IBM SPSS: IBM SPSS is an data analysis software usually use for social science research. It is available from IBM Corp. (2021). https://www.ibm.com/support/pages/downloading-ibm-spss-statistics-27

JASP: JASP is an open-source statistical software for data analysis which can perform similar functionalities like SPSS. It is freely available at https://jasp-stats.org
