# Supplementary material for: Teachers’ and students’ use of ChatGPT at Social science faculty in the public and private Universities of Bangladesh
Source: F1000Res. 2025 Sep 5;14:269. Originally published 2025 Mar 6. [Version 2] doi: 10.12688/f1000research.161351.2 (PMC12461191; doi:10.12688/f1000research.161351.2)
Supplement: Peer review history

# Reviewer report: Reviewer response for version 2

**DOI:** 10.5256/f1000research.187222.r411881

**Published:** 2025-09-24

**Authors:** Sarin Sok

**Affiliations:** 1 University of Puthisastra,, Phnom Penh, Cambodia

Thank you for giving me the chance to review this manuscript again. Overall, the authors addressed all my comments and suggestions

 With that being said, I came up with a few suggestions. For example, in Figure 2, page 16, some capital letters were not consistently used, so please pay closed attention on this matter. Another observation is related to the use of participants' quotes following the APA 7 style. Please kindly consider that quotation marks are not required to use when a quote is more than 40 words while you enter it. However, I suggest checking the journal's format.

Is the work clearly and accurately presented and does it cite the current literature?

Yes

If applicable, is the statistical analysis and its interpretation appropriate?

Yes

Are all the source data underlying the results available to ensure full reproducibility?

Yes

Is the study design appropriate and is the work technically sound?

Yes

Are the conclusions drawn adequately supported by the results?

Yes

Are sufficient details of methods and analysis provided to allow replication by others?

Partly

Reviewer Expertise:

AI in Education and research

I confirm that I have read this submission and believe that I have an appropriate level of expertise to confirm that it is of an acceptable scientific standard.

## References

[1] : Teachers’ and students’ use of ChatGPT at Social science faculty in the public and private Universities of Bangladesh. *F1000Research* .2025;14: 10.12688/f1000research.161351.2 10.12688/f1000research.161351.2 40657179

# Reviewer report: Reviewer response for version 2

**DOI:** 10.5256/f1000research.187222.r411882

**Published:** 2025-09-16

**Authors:** Soha Rawas

**Affiliations:** 1 Beirut Arab University, Beirut, Lebanon

I have reviewed the revisions and confirm that the authors have sufficiently addressed the previous comments.

Is the work clearly and accurately presented and does it cite the current literature?

Partly

If applicable, is the statistical analysis and its interpretation appropriate?

Yes

Are all the source data underlying the results available to ensure full reproducibility?

Partly

Is the study design appropriate and is the work technically sound?

Partly

Are the conclusions drawn adequately supported by the results?

Partly

Are sufficient details of methods and analysis provided to allow replication by others?

Partly

Reviewer Expertise:

Artificial Intelligence in Education, Educational Technology, Human–Computer Interaction, Ethical Implications of AI, Generative AI in Higher Education

I confirm that I have read this submission and believe that I have an appropriate level of expertise to confirm that it is of an acceptable scientific standard.

# Reviewer report: Reviewer response for version 1

**DOI:** 10.5256/f1000research.177362.r392651

**Published:** 2025-08-19

**Authors:** Soha Rawas

**Affiliations:** 1 Beirut Arab University, Beirut, Lebanon

**Summary of the Article**

 This study investigates how teachers and students at the social science faculties of public and private universities in Bangladesh are adopting and using ChatGPT in their academic practices. The authors employ a mixed-method approach framed by the UTAUT model, incorporating a large-scale survey (n=783) and in-depth interviews (n=32). The findings explore users’ perceptions, usage patterns, and factors influencing adoption, including performance expectancy, effort expectancy, and social influence.

**Major Comments**

**1. Clarity and Literature Review – *Partly***

 While the topic is timely and relevant, the manuscript suffers from **significant grammatical issues**, typographical errors, and frequent awkward phrasing. These linguistic issues hinder clarity and reduce the academic quality expected of a high-tier journal.

 Moreover, the literature review is somewhat **surface-level and descriptive**, lacking **critical engagement** with the most recent or global literature on GenAI tools in higher education. Key contributions from recent global studies (post-2023) on ChatGPT in education are missing.

**Recommendation**: The manuscript requires professional language editing and a more nuanced engagement with recent, high-impact literature to enhance clarity and academic positioning.

**2. Study Design and Technical Rigor – *Partly***

 The use of the UTAUT framework is theoretically sound. However, the authors do not use **validated scales** from prior UTAUT studies nor mention any **confirmatory factor analysis** to test construct validity. The convenience sampling for interviews is also not justified, and the **qualitative analysis is underdeveloped**, lacking direct quotes or clear thematic coding schemes.

**Recommendation**: Clarify how UTAUT items were developed or adapted. Strengthen qualitative analysis with detailed coding strategy and supporting quotations from participants.

**3. Methodological Transparency and Replicability – *Partly***

 The study reports basic details on questionnaire construction and Cronbach’s alpha, but omits critical information such as **survey instrument appendices**, **exact question wording**, and **full interview protocols**. These omissions hinder replication.

**Recommendation**: Include the full survey and interview protocols as supplementary material and provide examples of qualitative questions and coding categories.

**4. Statistical Analysis – *Yes***

 The statistical analysis is generally appropriate and clearly presented. Descriptive statistics, ANOVA, and Tukey HSD post hoc tests are suitable given the design. However, interpretation lacks **depth**, particularly in terms of effect size or practical implications.

**Recommendation**: Discuss the practical significance (not just statistical) of the reported group differences.

**5. Source Data Availability – *Partly***

 The manuscript mentions that data were cleaned and analyzed in SPSS, but does not **provide raw datasets or a link to the repository**, nor specify whether the qualitative transcripts are available (even partially).

**Recommendation**: Make anonymized survey and interview data available in a trusted open-access repository to ensure reproducibility.

**6. Conclusions – *Partly***

 While the conclusions are consistent with the data, they remain **descriptive and context-specific**, lacking deeper theoretical or cross-national implications. Ethical concerns are mentioned but not integrated meaningfully into the conclusions.

**Recommendation**: Expand the conclusion to reflect broader educational implications, policy insights, and integration with global literature on GenAI ethics in education.

**Summary of Required Revisions**

- Conduct full **language editing** to improve readability.
- Integrate **recent literature (2023–2025)** on ChatGPT and AI in education.
- Clarify and **justify UTAUT adaptation**, and provide survey and interview tools.
- Enrich the **qualitative analysis** with quotes and thematic clarity.
- Ensure **open access to raw data or codebooks** to support reproducibility.
- Provide more **theory-driven and generalizable conclusions**.

**Overall Assessment**

 This manuscript presents valuable empirical insights from an underrepresented geographic context. However, to meet the standards of a Q1 journal or rigorous peer-reviewed outlet, it must undergo **major revisions in language, methodological transparency, and analytical depth**

Is the work clearly and accurately presented and does it cite the current literature?

Partly

If applicable, is the statistical analysis and its interpretation appropriate?

Yes

Are all the source data underlying the results available to ensure full reproducibility?

Partly

Is the study design appropriate and is the work technically sound?

Partly

Are the conclusions drawn adequately supported by the results?

Partly

Are sufficient details of methods and analysis provided to allow replication by others?

Partly

Reviewer Expertise:

Artificial Intelligence in Education, Educational Technology, Human–Computer Interaction, Ethical Implications of AI, Generative AI in Higher Education

I confirm that I have read this submission and believe that I have an appropriate level of expertise to confirm that it is of an acceptable scientific standard, however I have significant reservations, as outlined above.

## References

[1] : Teachers’ and students’ use of ChatGPT at Social science faculty in the public and private Universities of Bangladesh. *F1000Research* .2025;14: 10.12688/f1000research.161351.1 10.12688/f1000research.161351.1 40657179

# Response

**Published:** 2025-08-26

**Authors:** Arifur Rahman

**Affiliations:** Institute of Education and Res, University of Dhaka, Dhaka, Dhaka, Bangladesh

Thank you very much for your valuable suggestions. In following, what, how and where we addressed your insightful suggestions carefully has been provided. 

**1. Clarity and Literature Review**

 While the topic is timely and relevant, the manuscript suffers from significant grammatical issues, typographical errors, and frequent awkward phrasing. These linguistic issues hinder clarity and reduce the academic quality expected of a high-tier journal.

 Moreover, the literature review is somewhat surface-level and descriptive, lacking critical engagement with the most recent or global literature on GenAI tools in higher education. Key contributions from recent global studies (post-2023) on ChatGPT in education are missing.

 Recommendation: The manuscript requires professional language editing and a more nuanced engagement with recent, high-impact literature to enhance clarity and academic positioning.

**Addressed Comment:** Thank you for your valuable suggestions.

 We have completed language editing of full manuscript considering grammatical issues, typographical errors, and awkward phrasing. Page 1-33.

 Following your instructions,  we have added a literature review section and used recent (post -2023), high impact literature in the ‘ Literature Review’ section  to enhance clarity and academic positioning. Page 3-6. 

**2. Study Design and Technical Rigor**

 The use of the UTAUT framework is theoretically sound. However, the authors do not use validated scales from prior UTAUT studies nor mention any confirmatory factor analysis to test construct validity. The convenience sampling for interviews is also not justified, and the qualitative analysis is underdeveloped, lacking direct quotes or clear thematic coding schemes.

 Recommendation: Clarify how UTAUT items were developed or adapted. Strengthen qualitative analysis with detailed coding strategy and supporting quotations from participants.

**Addressed Comment:** Thank you for your suggestion.

 We have explained   how UTAUT items were developed or adapted. We have also strengthened qualitative analysis of data. Detailed coding strategy have been explained and more quotations from the participants has been added

 Page- 7, 8, 9, and finding section

**3. Methodological Transparency and Replicability**

 The study reports basic details on questionnaire construction and Cronbach’s alpha, but omits critical information such as survey instrument appendices, exact question wording, and full interview protocols. These omissions hinder replication.

 Recommendation: Include the full survey and interview protocols as supplementary material and provide examples of qualitative questions.

**Addressed Comment:** Thank you very much. Full survey questionnaires for teachers and students, and interview protocols for qualitative data were added in the Appendix and depository linked was provided in the article.

**4. Statistical Analysis**

 The statistical analysis is generally appropriate and clearly presented. Descriptive statistics, ANOVA, and Tukey HSD post hoc tests are suitable given the design. However, interpretation lacks depth, particularly in terms of effect size or practical implications.

 Recommendation: Discuss the practical significance (not just statistical) of the reported group differences.

**Addressed Comment:** Thank you again. The statistical findings include group differences and practical significance. Your suggestions have been addressed in Page 16, 18, 19  20, 21.

**5. Source Data Availability**

 The manuscript mentions that data were cleaned and analyzed in SPSS, but does not provide raw datasets or a link to the repository, nor specify whether the qualitative transcripts are available (even partially).

 Recommendation: Make anonymized survey and interview data available in a trusted open-access repository to ensure reproducibility.

**Addressed Comment: **Thank you very much. Data repository linked was provided from where anyone can download data.

**6. Conclusions**

 While the conclusions are consistent with the data, they remain descriptive and context-specific, lacking deeper theoretical or cross-national implications. Ethical concerns are mentioned but not integrated meaningfully into the conclusions.

 Recommendation: Expand the conclusion to reflect broader educational implications, policy insights, and integration with global literature on GenAI ethics in education.

**Addressed Comment: **Thank you for your valuable suggestions. We have revised the ‘conclusion’ and ethical concerns were effectively integrated into the conclusion, along with broader impact, policy, and GenAI ethics in education.  Page 31, 32.

# Reviewer report: Reviewer response for version 1

**DOI:** 10.5256/f1000research.177362.r373476

**Published:** 2025-04-21

**Authors:** Sarin Sok

**Affiliations:** 1 University of Puthisastra,, Phnom Penh, Cambodia

Dear Editor, 

 Thank you so much for giving me an opportunity to review this manuscript. While this research topic is emerging and significant for global readers, and the paper is well-structured and academically sound, some issues were identified, and that they must be addressed provide nuanced insights and clarity. 

**Introduction**: 

 In this introduction part, it clearly and critically discussed the use of ChatGPT in the global and local contexts. However, it would be of benefit to combine the last sentence of the last para with second one. E.g., this study aims to address the significant research gap by investigating …….in Bangladesh. 

 Moreover, although concise research aim was highlighted, it might be beneficial to clearly mention your research questions. This will allow readers to better understand to findings. 

**Research methods**

 The research methods were clearly discussed, but lack of justification to some extent. Therefore, it is important to address these concerns: How were the questionnaires established? They were informed by synthesising relevant existing studies or adopted from other ones? 

 It also appeared to me that you tended to miss discussing how to ensure the validity of your questionnaire beyond the Cronbach’s alpha. 

 It is also important to comment on what you did after conducting the pilot study with teachers and students.  Did you revise the questionnaires? Were the responses in the pilot used included in the current study? 

 With regard to questionnaire distribution, How did you distribute this questionnaire? What tool/platform did you use, Google Forms or .....? 

 Please also comment the number of female teachers and students. 

**Findings**

 It is of benefit to provide the table of participants's profile in the qualitative stage, as it may enable readers to understand their profile. 

 Beyond the saying a public or private student, can clearly specify the participants whom you quoted in your findings. In this regard, you may use S1, S1….S16 for students, and T1, T2…T16 for teachers. This may provide better detail for the readers. 

**Conclusion**

 While conclusion, implications, and limitations were clearly and comprehensively written. It might be better sound and more logical to include these implications and limitations in the conclusion section, probably in the last para. In particular, the limitations should like to call for future research to address these limitations. 

 Thank you.

Is the work clearly and accurately presented and does it cite the current literature?

Yes

If applicable, is the statistical analysis and its interpretation appropriate?

Yes

Are all the source data underlying the results available to ensure full reproducibility?

Yes

Is the study design appropriate and is the work technically sound?

Yes

Are the conclusions drawn adequately supported by the results?

Yes

Are sufficient details of methods and analysis provided to allow replication by others?

Partly

Reviewer Expertise:

AI in Education and Research, MOOCs for Professional Development, and Teacher's Professional Development

I confirm that I have read this submission and believe that I have an appropriate level of expertise to confirm that it is of an acceptable scientific standard, however I have significant reservations, as outlined above.

# Response

**Published:** 2025-08-26

**Authors:** Arifur Rahman

**Affiliations:** Institute of Education and Res, University of Dhaka, Dhaka, Dhaka, Bangladesh

**Thank you very much for your valuable suggestions. In following, how and where we addressed your insightful comments are provided.**

**Introduction:**

 In this introduction part, it clearly and critically discussed the use of ChatGPT in the global and local contexts. However, it would be of benefit to combine the last sentence of the last para with second one. E.g., this study aims to address the significant research gap by investigating …….in Bangladesh.

 Moreover, although concise research aim was highlighted, it might be beneficial to clearly mention your research questions. This will allow readers to better understand to findings.

**Address Comment**: Thank you very much for your valuable suggestion. We have addressed it now. Page- 3. In the introduction section, the last paragraph has been reorganized according to the suggestions, along with the addition of research questions.

**Research methods**

 The research methods were clearly discussed, but lack of justification to some extent. Therefore, it is important to address these concerns: How were the questionnaires established? They were informed by synthesising relevant existing studies or adopted from other ones?

 It also appeared to me that you tended to miss discussing how to ensure the validity of your questionnaire beyond the Cronbach’s alpha.

 It is also important to comment on what you did after conducting the pilot study with teachers and students.  Did you revise the questionnaires? Were the responses in the pilot used included in the current study?

 With regard to questionnaire distribution, How did you distribute this questionnaire? What tool/platform did you use, Google Forms or .....?

 Please also comment the number of female teachers and students. to be briefly explained for clarity.

**Addressed Comment: **Thank you very much for your suggestion. We have addressed it now. Page-7, 8. Justification for the research tools guiding the UTAUT model and existing literature was provided. Moreover, content validity and reflection from the pilot test have been added. Questionnaire distribution for data collection was added, and the number of female participants was provided for clarity.

**Findings**

 It is of benefit to provide the table of participants's profile in the qualitative stage, as it may enable readers to understand their profile.

 Beyond the saying a public or private student, can clearly specify the participants whom you quoted in your findings. In this regard, you may use S1, S1….S16 for students, and T1, T2…T16 for teachers. This may provide better detail for the readers.

**Addressed Comment:** Thank you for this suggestion. We have addressed it now. We have provided profile of participants and used S1, S2, S3....for students and T1, T2, T3 ....for teachers. Page 21, 22, 23.

**Conclusion**

 While conclusion, implications, and limitations were clearly and comprehensively written. It might be better sound and more logical to include these implications and limitations in the conclusion section, probably in the last para. In particular, the limitations should like to call for future research to address these limitations.

**Addressed Comment:** Thank you again for your valuable suggestions.  We have deleted the sections ‘implications’ and ‘limitations’. We have included these two sections in the last part of ‘conclusion’ a suggested.  Page 31. 32, 33.
